# Effects of Nutritional Interventions during Pregnancy on Infant and Child Cognitive Outcomes: A Systematic Review and Meta-Analysis

**DOI:** 10.3390/nu9111265

**Published:** 2017-11-20

**Authors:** Rachael M. Taylor, Shanna M. Fealy, Alessandra Bisquera, Roger Smith, Clare E. Collins, Tiffany-Jane Evans, Alexis J. Hure

**Affiliations:** 1 Priority Research Centre for Reproductive Science, University of Newcastle, Callaghan, NSW 2308, Australia; roger.smith@newcastle.edu.au; 2 Faculty of Health and Medicine, School of Medicine and Public Health, University of Newcastle, Callaghan, NSW 2308, Australia; shanna.fealy@newcastle.edu.au (S.M.F.); alexis.hure@newcastle.edu.au (A.J.H.); 3 Hunter Medical Research Institute, 1 Kookaburra Circuit, New Lambton Heights, NSW 2305, Australia; alessandra.bisquera@kcl.ac.uk (A.B.); clare.collins@newcastle.edu.au (C.E.C.); tiffany.evans@hmri.org.au (T.-J.E.); 4 Faculty of Health and Medicine, School of Nursing & Midwifery, University of Newcastle, Callaghan, NSW 2308, Australia; 5 Clinical Research Design IT and Statistical Support (CReDITSS) Unit, Hunter Medical Research Institute, 1 Kookaburra Circuit, New Lambton Heights, NSW 2305, Australia; 6 Faculty of Health and Medicine, School of Health Sciences, University of Newcastle, Callaghan, NSW 2308, Australia; 7 Priority Research Centre in Physical Activity and Nutrition, University of Newcastle, Callaghan, NSW 2308, Australia; 8 Priority Research Centre for Gender, Health and Ageing, University of Newcastle, Callaghan, NSW 2308, Australia

**Keywords:** behaviour, child, cognition, cognitive function, infant, nutrition, pregnancy, supplement

## Abstract

Background: Epidemiological studies have demonstrated that folate, iodine and iron intake during pregnancy impacts on foetal brain development and cognitive function. However, in human studies, the relationship with other dietary nutrients is less clear. Objective: This systematic review aims to critically appraise the current literature and meta-analyses results from nutritional interventions during pregnancy that aimed to optimise infant and child cognitive outcomes. Design: Ten electronic databases were searched for articles published up to August 2017. The search was limited to articles published in English. Randomised controlled trials (RCTs) testing the impact of any nutritional intervention (dietary counselling, education, nutrient supplementation, fortified foods and/or foods) during pregnancy on cognitive outcomes of children (<10 years old). Two independent reviewers assessed study eligibility and quality using the American Dietetic Association quality criteria checklist for primary research. Standardised mean differences were used for nine cognitive domains to measure effects for meta-analyses. Results: A total of 34 RCTs were included (21 studies included children aged less than 35 months, 10 studies included children aged 36–60 months and 3 studies included children aged 61–119 months). The types of nutritional interventions included nutrient supplements, whole foods, fortified foods and nutrition education. The following nine cognition outcomes: attention, behaviour, crystallised intelligence, fluid intelligence, global cognition, memory, motor skills, visual processing, and problem solving were not significantly impacted by nutritional interventions, although 65% of studies conducted post-hoc data analyses and were likely to be underpowered. Although, long chain polyunsaturated fatty acids (LCPUFA) supplementation was associated with a marginal increase in crystallised intelligence (Effect size (ES): 0.25; 95% confidence interval (95% CI): −0.04, 0.53), the effect was not statistically significant (*p* = 0.09), with significant study heterogeneity (*p* = 0.00). Conclusions: LCPUFA supplementation may be associated with an improvement in child crystallised intelligence, however further research is warranted. The remaining eight cognition domains were not significantly impacted by maternal nutritional interventions.

## 1. Introduction

Adequate nutrition during the prenatal period and early years of life is essential for brain development and cognitive function. Approximately 28 days after conception the neural plate folds and fuses, forming the neural tube, which gives rise to the development of the foetal brain [1]. Adequate folate from the maternal diet during this period is essential for the formation of the neural tube, a deficiency in this nutrient can adversely affect brain development, resulting in neural tube defects, spina bifida and encephalocoele [2,3,4,5,6]. Following the formation of the neural tube, neurodevelopmental processes including, cell proliferation and migration occur during gestation, while neurogenesis, synaptic formation and myelination continue until late adolescence [7]. Iodine is necessary for neural cell migration and differentiation, synaptogenesis and myelination [8], while dietary iron is necessary for neurogenesis and dopamine production [9,10,11]. Deficiency in these nutrients are known to compromise brain development and cause significant cognitive impairment in the offspring [8,12]. The importance of an adequate intake of folate, iodine and iron during pregnancy for foetal brain development has been well explored; however, the developmental role of other dietary nutrients (e.g., zinc, long-chain polyunsaturated fatty acids (LCPUFAs)) remains unclear from human studies. 

The link between early-life nutrition and child cognitive function is difficult to establish because the brain is a heterogeneous organ consisting of multiple anatomic regions (e.g., the hippocampus, striatum, cortex) and neurodevelopmental processes (e.g., synaptogenesis, myelination) with distinct developmental trajectories that span and peak at different times [13,14]. For example, myelination commences at 12–14th week of gestation and occurs at a peak rate during the first two years of life, but continues until adulthood [15]. The prefrontal cortex, which has a prominent role in higher cognitive control, develops in growth spurts during the first two years of life, once again between seven and nine years of age and also at approximately 15 years of age [16,17]. The effect of nutrition on cognitive function depends on the nutrients involved and the maturation stage of the brain. These nutrient effects on brain structure and function may occur in the short-term, while others may not be apparent until full maturation is reached [18]. 

Optimising brain development and cognitive function holds major long-term consequences for individuals and societies. Cognitive function affects academic performance and the level of education attained [19]. Higher educational attainment is related to a lower burden of disease, due to greater health care access and healthy lifestyle behaviours [20]. Suboptimal cognition during childhood has been associated with an increased risk of adolescent delinquency [21,22,23] and adult violent criminality [21,24,25,26]. Incarceration can cause permanent cognitive damage, which holds major implications for future education, employment and health [27]. Strategies that aim to maximise the cognitive performance of children are therefore important for public health policy and nutrition during pregnancy. 

This systematic review and meta-analysis aimed to determine whether nutritional intervention/s during pregnancy alter cognitive outcomes during infancy and late childhood. Larson et al. [28] analysed the impact of maternal nutritional interventions on child cognition, however this review focused on short-term cognitive outcomes in children under the age of two years in developing countries. Therefore, a systematic review that considers the impact of maternal nutritional interventions on long-term cognitive outcomes of children living in developed and developing countries is warranted to provide the most comprehensive evaluation of the literature in this area.

## 2. Methods

The review protocol was developed using The Cochrane Handbook for Systematic Reviews of Interventions. The Preferred Reporting Items for Systematic Review Meta-Analyses (PRISMA) [29] was applied. Similar methods have been followed previously by Gresham et al. [30]. The review protocol can be found in Supplementary Figure S1.

### 2.1. Search Strategy

A search strategy was devised with the assistance of a medical research librarian (DB). The final electronic literature search was conducted on August 2017, without date limits, using Medline, Pre-Medline, Embase, PsycInfo and Maternity and Infant Care via Ovid (http://www.ovid.com/), Scopus (http://www.scopus.com/), Proquest (http://www.proquest.com/), Web of Science (http://apps.webofknowledge.com) and Cumulative Index to Nursing and Allied Health Literature via EBSCO (http://www.ebsco.com/cinahl). The Cochrane Library was also searched separately to identify any similar systematic reviews that have been conducted previously. The following keywords were used: pregnan*, cognit*, neurodevelopment, infant*, child*, randomised control* trial, clinical trial, food* nutrition and supplement*. Full details of the search strategy tailored for each database can be found in Supplementary Table S1. The Boolean operation (i.e., AND, OR) was used to combine keywords that were searched as free text in the title, abstract or topics from all papers. All searches were limited to human studies published in English and citations were downloaded into the reference manager program ENDNOTE X6.v (New York, NY, USA: Thomson Reuters 2012). The keywords: mental development and motor development, were searched in all databases separately, to ensure that no eligible studies were missed from the initial search strategy. 

### 2.2. Study Selection

Eligibility of the retrieved publications was assessed by two independent reviewers (RMT and SMF) as recommended by PRISMA [29]. Publication title and abstracts were evaluated against the inclusion and exclusion criteria (Table 1) Publications were excluded by a hierarchical approach based on the study design, population, intervention and outcomes. Publications meeting the initial eligibility screening were retrieved in full and were then subjected to a second round of screening by the same two reviewers to determine the final inclusion or exclusion. Assessment discrepancies between reviewers were resolved by discussion or assessment by a third party independent reviewer (AJH). The reference lists of the included publications were searched separately to identify any relevant articles that were not detected by the electronic search strategy. The abstract and then the full texts were retrieved for consideration. After the retrieval of the full text a final decision was made about the eligibility of the publication by the first reviewer (RMT). 

### 2.3. Eligibility Criteria

Table 1 outlines the inclusion and exclusion criteria for this systematic review. Studies that delivered nutritional interventions to the mother during pregnancy (exclusively) or during pregnancy plus the post-natal period (<10 years) were included. This review included studies that measured cognition in children up to nine years of age because the effect of nutritional interventions may not be apparent until later life [18]. In addition, cognition assessment tests in older children (>7 years) are a more reliable predictor of adult cognitive function [31]. Nutritional interventions that commenced after pregnancy were excluded. This review included studies that measured cognition (as a primary or secondary outcome) in infants and young children using one or more cognition assessment tests or subtests. Cognition domains were based on the Cattell−Horn−Carroll (CHC) Theory of Intelligence [32]. The domains fluid and crystallised intelligence, specified in the CHC model [32], were combined to form the global cognition domain for cognition assessment tests that reported an overall global composite score. Verbal composite scores were grouped in the crystallised intelligence domain and non-verbal composite scores were grouped in the fluid intelligence domain. Short-term memory and long-term retrieval domains were combined to form an overall memory domain. The remaining six cognition domains of the CHC model [32] (quantitative reasoning, reading and writing ability, visual processing, auditory processing, processing speed and correct/decision speed) were included. Attention, motor skills and behaviour were also analysed because these domains are commonly analysed in cognition assessment tests. For example, the Intergrowth-21st Neurodevelopment Assessment reports child cognitive scores for the domains attention, motor skills and behaviour [33]. In addition, Bayley Scales of Infant development (third edition) includes a behaviour rating scale, which measures behavioural factors including orientation/engagement and emotional regulation [34]. This review included the outcome visual processing that focuses on both the response and comprehension of visual stimuli. Visual acuity which primarily focuses on vision clarity, was not reported as an outcome in this review. Similar methods have been described previously by Eilander et al. [35]. 

### 2.4. Quality Assessment

Articles considered eligible were assessed for methodological quality in their reporting using the American Dietetic Association (ADA) Quality Criteria Checklist for Primary Research [36] which is applicable to evaluating the validity of nutrition studies. The quality of the articles was assessed by two independent reviewers (RMT and SMF) and assessment discrepancies between reviewers, were managed through discussion or resolved by the third independent reviewer (AJH). The Quality Criteria Checklist [36] rates the study design and execution, as well as the risk of bias using ten validity questions, including four priority questions which must be satisfactory to gain a positive rating. Based on the responses to these questions, the checklist assigns a quality rating: positive (answered “yes” to six or more validity questions, including all four priority questions), negative (answered “no” to six or more validity questions) or neutral (answered “no” to one or more of the four priority criteria questions) [36]. No studies were given a negative rating in this review, therefore no study exclusions were made. 

### 2.5. Data Extraction

The first reviewer (RMT) extracted relevant data from the included studies using an Excel spreadsheet. The Organisation for Economic Co-operation and Development (OECD) criteria [37] were used to classify country income into four categories: low, lower-middle, higher-middle and high. Nutritional interventions were divided into six categories; single nutrient supplement, multiple micronutrient supplement, fortified foods, foods, supplements with foods, supplements with foods and nutrition education or no nutrition intervention (control group). For the purpose of this review a multiple micronutrient supplement was defined as two or more nutrients which is consistent with other authors [38,39,40]. The cognition outcomes data included the mean, standard deviation, confidence intervals, minimum and maximum range and odds ratio. Corresponding authors were emailed if additional data or clarification was required. 

### 2.6. Data Synthesis

The nutritional interventions provided across the included studies were grouped together according to the nutrient supplied and the type of intervention (i.e., supplement, dietary changes). The cognition outcomes reported were organised into related cognition domains and the type of scoring (i.e., subtest or composite score). Thirteen cognition domain categories were derived: (i) fluid intelligence; (ii) crystallised intelligence; (iii) quantitative knowledge; (iv) memory; (v) visual processing; (vi) auditory processing; (vii) processing speed; (viii) correct decision speed; (ix) reading and writing; (x) global cognition; (xi) attention; (xii) motor skills; (xiii) behaviour. 

### 2.7. Statistical Analysis

The aim of meta-analysis was to present a single effect size for the 13 specified cognition scores. The standardised mean difference (SMD) was used to measure the effect considering that all outcomes were measured using different cognition assessment tests and reporting units. The SMD, also known as Cohen’s d, is defined as the difference between the mean of the intervention and control group divided by the standard deviation of the data. Methods to convert other units (e.g., Odds ratio) to SMD are described in Sanchez-Meca et al. [41]. Studies that presented multiple cognition outcomes within the same cognition domain, multiple time-points, or multiple interventions for multiple micronutrient supplementation with only a single control group, the respective method described by Borenstein et al. [42] was used to calculate a summary SMD and standard error which accounts for non-independence within each study. As the correlations between outcomes were not reported by the studies, the correlation was assumed to be 0.5, and sensitivity analyses were performed using a higher (0.8) or lower (0.2) correlation to assess the changes in the standard error.

Supplementary Table S2 shows an example of how the composite effect size was created. A random effects meta-analytic model (using the method of DerSimonian and Laird [43]) was conducted for each cognitive outcome, where the observed effect sizes are regarded to vary due to sampling variance. A fixed effects model using inverse variance weights was also conducted for secondary analysis. The heterogeneity and total variation of studies were analysed using the *I*_2_ statistic [44]. Funnel plots were used to assess publication bias. Subgroup analysis was performed for each outcome by the nutrient of interest. A sensitivity analysis with a random effects model was conducted in which the studies were stratified by country income. The statistical analysis was conducted using the metan command in the statistical software package STATA 13 (College Station, TX, USA: StataCorp LP 2013).

## 3. Results

### 3.1. Search Results

**Figure 1:**
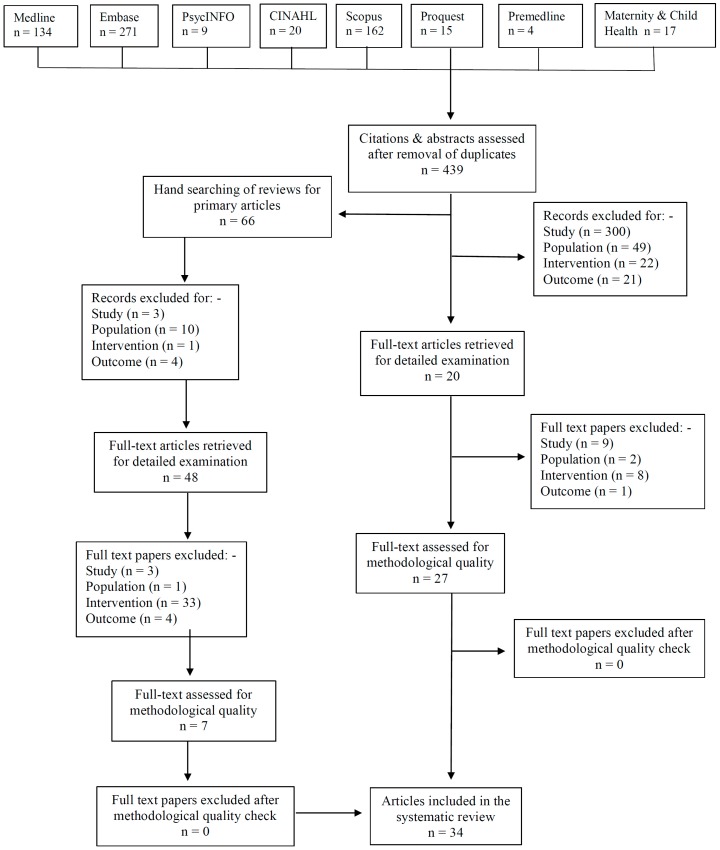
Flow chart for study selection process.

A total of 1195 publications (excluding duplicates, *n* = 439) were assessed against the inclusion and exclusion criteria (Figure 1). Seven included publications were identified from hand searching the reference lists of included publications detected from the initial search strategy. These articles all used the keyword mental development, which was not included separately. This systematic review resulted in 34 included publications after inclusion and exclusion criteria were applied to full texts. 

### 3.2. Quality

A summary of the ADA quality assessment including participant selection, handling of withdrawals and the use of study blinding for each trial is presented in Supplementary Table S3. Direct (e.g., blood, urine tests) intervention compliance measures were used in 12 out of 34 studies [45,46,47,48,49,50,51,52,53,54,55,56]. Indirect (e.g., pill count, self-reported) intervention compliance measures were used in 15 out of 34 studies [57,58,59,60,61,62,63,64,65,66,67,68,69,70,71]. The attrition rate ranged from 0–99% indicating the potential for bias with a loss to follow-up. Post-hoc data analysis was used in 22 studies [46,47,49,50,51,53,55,56,57,58,60,61,63,64,65,66,67,69,72,73,74,75]. 

### 3.3. Description of Studies

Table 2 summarises the characteristics of the included studies. The earliest publication was from 1979 [76] and the latest from 2017 [72,75]. A summary of the studies from the OECD is provided in Supplementary Table S5. In summary, 50% of studies were from high-income countries, 29% of studies were from middle-income countries and 21% of studies were from low-income countries.

### 3.4. Participants

The study populations were mostly healthy pregnant women, except one publication [61] which included pregnant women with human immunodeficiency virus type 1 (HIV-1), although pregnant women were excluded if they were diagnosed with acquired immunodeficiency syndrome (AIDS) according to the World Health Organisation (WHO) definition [77]. 

### 3.5. Intervention

A summary of the nutritional interventions of the studies is provided in Supplementary Table S4. In summary, 76% of studies provided a nutrient supplement exclusively for the study intervention, no studies provided nutrition counselling or education as a stand-alone intervention. 

### 3.6. Cognitive Outcomes

Table 2 summarises the cognition assessment tools used in each study. Further details about the cognition assessment tests used is provided in Supplementary Table S5. The age of child cognitive testing ranged from less than two weeks [76] to nine years old [71], however 62% of studies (*n* = 13 trials) reported cognition outcomes in children less than 35 months, and 46% of these studies (*n* = 6 trials) provided LCPUFA interventions in high-income countries. Meta-analysis was completed for nine cognition outcomes measured in the 34 included studies. The analyses for each cognition domain is described below, however in summary all cognition domains were not significantly affected by nutritional interventions.

### 3.7. Outcome 1: Behaviour

**Figure 2:**
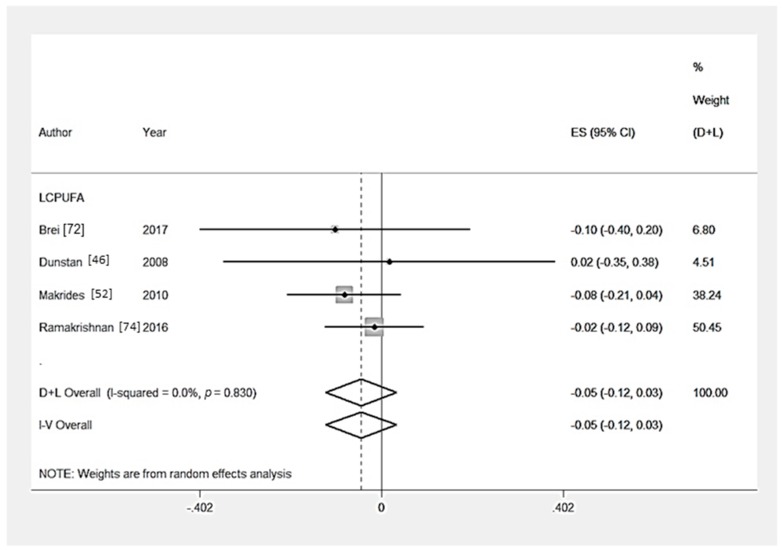
Forest plot for child behaviour outcomes. The overall effect size was estimated by standardised mean difference (SMD). LCPUFA, long chain polyunsaturated fatty acids. D+L, random-effects estimate (Der Simonian and Laird method) and I-V, fixed-effects estimate (inverse variance method).

Ten RCTs [46,48,52,53,57,64,65,70,72,74] measured the effect of a nutritional intervention on behaviour composite scores. Multiple outcomes were combined to create a composite score in Brei et al. [72] (2 outcomes), Makrides et al. [52] (2 outcomes), Ramakrishnan et al. [74] (4 outcomes), Zhou et al. [65] (5 outcomes), Zhou et al. [70] (2 outcomes), Caulfield et al. [57] (5 outcomes), and Dunstan et al. [46] (4 outcomes). There was no significant effect of LCPUFA supplementation (*n* = 4 trials) [46,52,72,74], multiple micronutrient supplementation (*n* = 1 trial) [53], iron supplementation (*n* = 1 trial) [65], iodine supplementation (*n* = 1 trial) [70], zinc supplementation (*n* = 1 trial) [57] or a food-based intervention (*n* = 1 trial) [64] (Table 3) (Table 3 and Figure 2). Hanieh et al. [48] investigated the effect of nutrient supplements of varying dosage on cognition, no significant effect on infant behaviour composite score was reported. From the statistical tests of Egger (*p* = 0.72), there was no evidence of publication bias (Supplementary Figure S2). From the sensitivity analysis, the country-income of the studies did not significantly (*p* > 0.05) affect child behaviour outcomes in studies that provided LCPUFA supplementation (Supplementary Figure S3). 

### 3.8. Outcome 2: Memory

Four RCTs [45,55,65,74] measured the effect of a nutritional intervention on memory composite score. There was no significant effect of iron supplementation (*n* = 1 trial) [55], zinc supplementation (*n* = 1 trial) [65], choline supplementation (*n* = 1 trial) [45] or LCPUFA supplementation (*n* = 1 trial) [74] on memory composite scores. 

### 3.9. Outcome 3: Motor Skills

**Figure 3:**
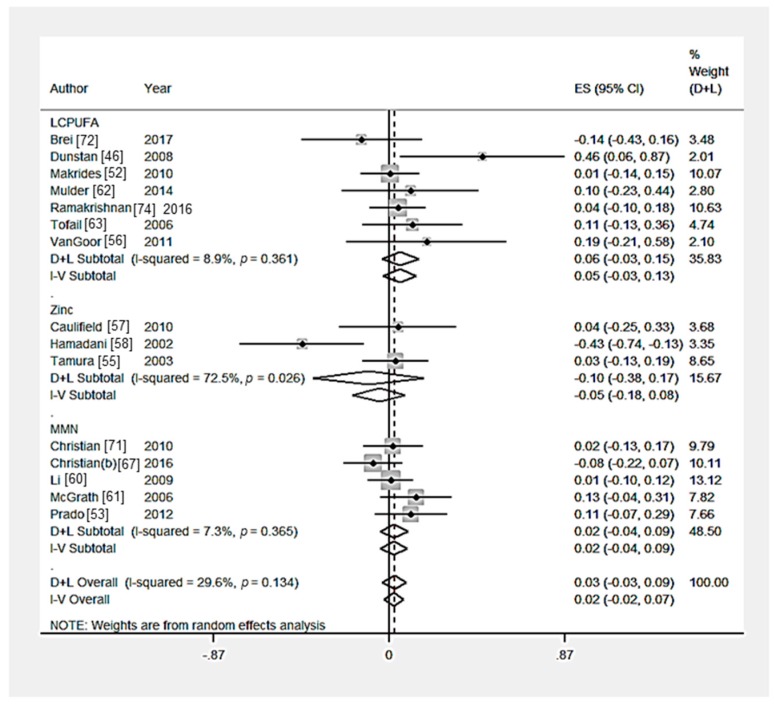
Forest plot for child motor skills outcomes. The overall effect size was estimated by standardised mean difference (SMD). LCPUFA, long chain polyunsaturated fatty acids; MMN, multiple micronutrient. D+L, random-effects estimate (Der Simonian and Laird method) and I-V, fixed-effects estimate (inverse variance method).

Eighteen RCTs [46,48,52,53,55,56,57,58,60,61,62,63,64,67,71,72,73,74] measured the effect of a nutritional intervention on the motor skills score. Multiple outcomes were combined to create a composite score in Brei et al. [72] (2 outcomes), Dunstan et al. [46] (2 outcomes), Mulder et al. [62] (2 outcomes), and Christian et al. [71] (2 outcomes). Li et al. [60] Chang et al. [73], and Christian et al. [71] used two interventions and one control group, the sample populations were not independent, therefore the effect sizes were combined. McGrath et al. [61] also used multiple independent intervention and control groups, therefore the effect sizes were kept separate. There was no significant effect of multiple micronutrient interventions (*n* = 6 trials) [53,60,61,67,71,73], LCPUFA supplementation (*n* = 7 trials) [46,52,56,62,63,72,74], zinc supplementation (*n* = 3 trials) [55,57,58] or food-based intervention (*n* = 1 trial) [64] on motor skills composite scores (Table 3 and Figure 3). Hanieh et al. [48] investigated the effect of nutrient supplementation of varying dosage on cognition, no significant effect on infant motor skills composite score was reported. The heterogeneity among the zinc intervention trials was significant (*p* = 0.03). From the statistical tests of Egger (*p* = 0.54), there was no evidence of publication bias (Supplementary Figure S4). The heterogeneity among the zinc intervention trials was significant (*p* = 0.03). From the sensitivity analysis, the country-income of the studies did not significantly (*p* > 0.05) affect child motor skills (Supplementary Figure S5). 

### 3.10. Outcome 4: Fluid Intelligence

**Figure 4:**
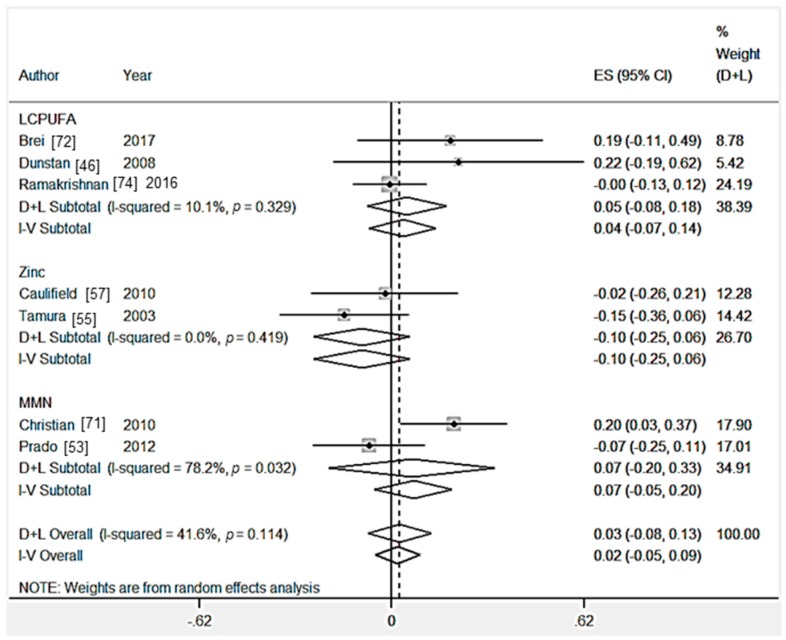
Forest plot for child fluid intelligence outcomes. The overall effect size was estimated by standardised mean difference (SMD). LCPUFA, long chain polyunsaturated fatty acids; MMN, multiple micronutrient. D+L, random-effects estimate (Der Simonian and Laird method) and I-V, fixed-effects estimate (inverse variance method).

Eight RCTs [46,53,55,65,71,72,74] measured the effect of a nutritional intervention on the fluid intelligence composite score. Multiple outcomes were combined to create a composite score in Brei et al. [72] (2 outcomes), and Ramakrishnan et al. [74] (2 outcomes).There was no significant effect of LCPUFA supplementation (*n* = 3 trials) [46,72,74], multiple micronutrient supplementation (*n* = 2 trials) [53,67], zinc supplementation (*n* = 2 trials) [55,57] or iron supplementation (*n* = 1 trial) [65] on fluid intelligence composite scores (Table 3 and Figure 4). From the statistical tests of Egger (*p* = 0.39), there was no evidence of publication bias (Supplementary Figure S6). From the sensitivity analysis, the country-income of the studies did not significantly (*p* > 0.05) affect child fluid intelligence (Supplementary Figure S7). 

### 3.11. Outcome 5: Global Cognition

**Figure 5:**
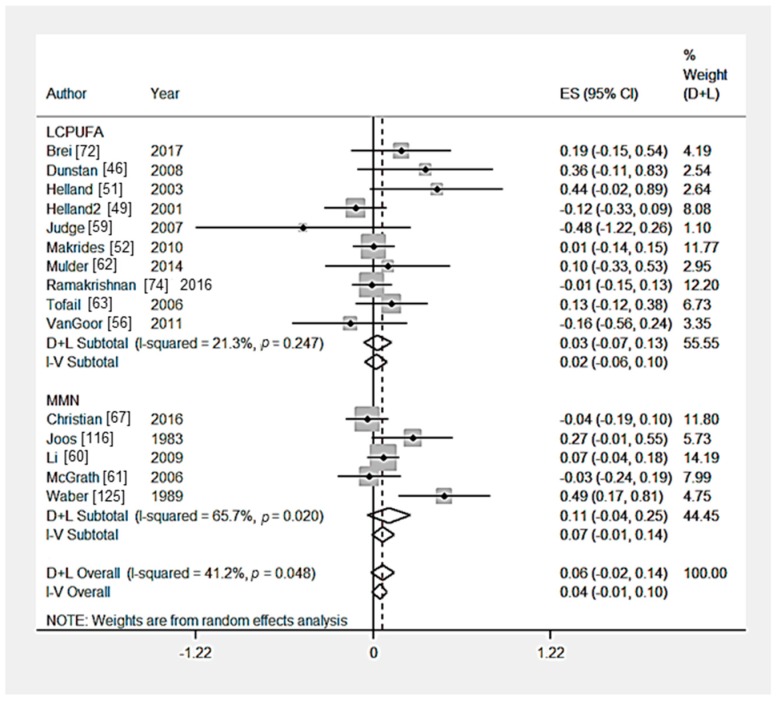
Forest plot for child global cognition outcomes. The overall effect size was estimated by standardised mean difference (SMD). LCPUFA, long chain polyunsaturated fatty acids; MMN, multiple micronutrient. D+L, random-effects estimate (Der Simonian and Laird method) and I-V, fixed-effects estimate (inverse variance method).

Twenty RCTs [46,48,49,51,52,56,57,59,60,61,62,63,65,67,69,72,73,74,116,125] measured the effect of a nutritional intervention on global cognition. The effect size of multiple time points were combined in Chang et al. [73], Helland et al. [49], Helland et al. [51] and Helland et al. [50]. Li et al. [60] Van Goor et al. [56] Chang et al. [73] and Waber et al. [125] used multiple interventions and one control group, the sample populations were not independent, therefore the effect sizes were combined. McGrath et al. [61] also used multiple independent intervention and control groups, therefore the effect sizes were kept separate. There was no significant effect of multiple micronutrient interventions (*n* = 6 trials) [60,61,67,73,116,125] LCPUFA interventions (*n* = 10 trials) [46,49,51,52,56,59,62,63,72,74] iron supplementation (*n* = 1 trial) [65] or vitamin A supplementation (*n* = 1 trial) [69] on global cognition composite scores (Table 3 and Figure 5). Hanieh et al. [48] investigated the effect of nutrient supplementation of varying dosage on cognition, iron and folic acid supplements given twice weekly was shown to significantly effect global cognition composite scores in infants at six months. The heterogeneity among the multiple micronutrient intervention trials was significant (*p* = 0.04). From the statistical tests of Egger (*p* = 0.82), there was no evidence of publication bias (Supplementary Figure S8). From the sensitivity analysis, the country-income of the studies did not significantly (*p* > 0.05) affect child global cognition outcomes (Supplementary Figure S9). 

### 3.12. Outcome 6: Crystallised Intelligence 

**Figure 6:**
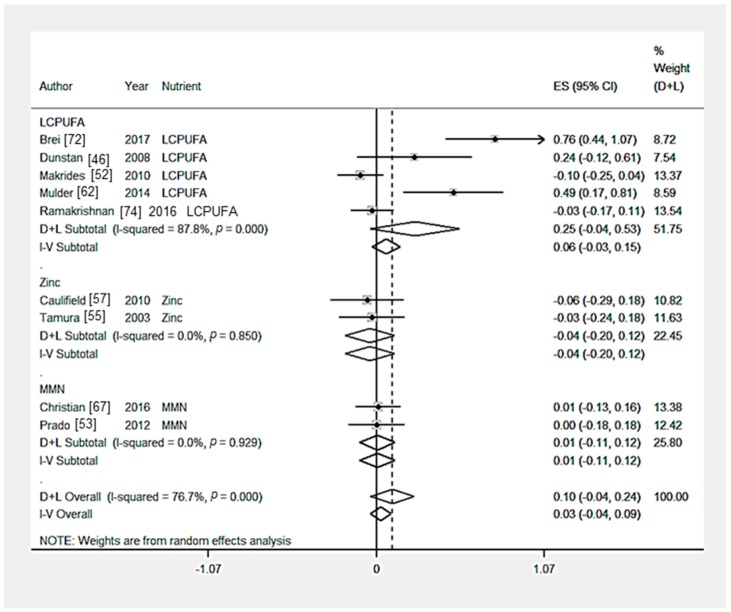
Forest plot for child crystallised intelligence outcomes. The overall effect size was estimated by standardised mean difference (SMD). LCPUFA, long chain polyunsaturated fatty acids; MMN, multiple micronutrient. D+L, random-effects estimate (Der Simonian and Laird method) and I-V, fixed-effects estimate (inverse variance method).

**Figure 7:**
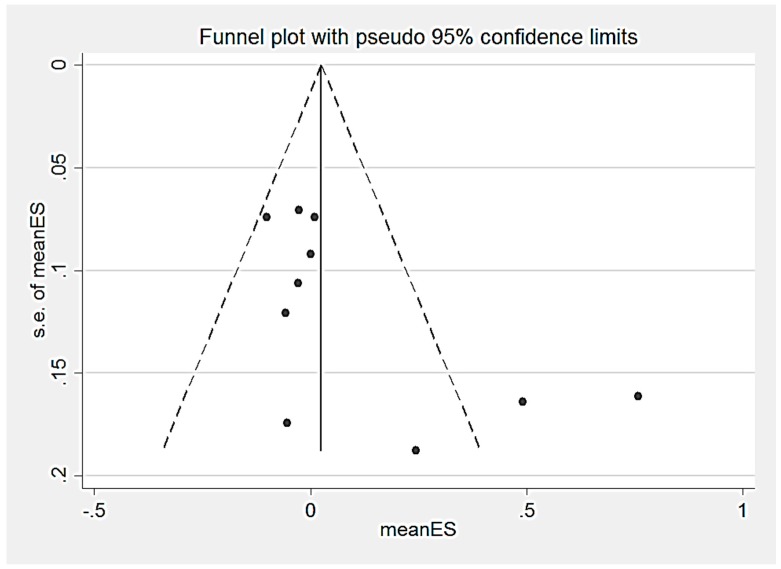
Funnel plot for child crystallized intelligence outcomes with 95% confidence limits. meanSE, mean standard error.

Eleven RCTs [46,48,52,53,55,57,62,65,67,72,74] measured the effect of a nutritional intervention on crystallised intelligence composite score. Multiple outcomes were combined to create a composite score in Brei et al. [72] (2 outcomes), Caulfield et al. [57] (3 outcomes), Dunstan et al. [46] (4 outcomes) and Mulder et al. [62] (7 outcomes). There was no significant effect of LCPUFA supplementation (*n* = 5 trials) [46,52,62,72,74], multiple micronutrient supplementation [53,67] (*n* = 2 trials), zinc supplementation (*n* = 2 trials) [55,57] or iron supplementation (*n* = 1 trial) [65] on crystallised intelligence composite scores (Table 3 and Figure 6). Hanieh et al. [48] investigated the effect of nutrient supplementation of varying dosage on cognition, no significant effect on child crystallised intelligence composite score was reported. The heterogeneity among the LCPUFA intervention trials was significant (*p* = 0.00). From the statistical tests of Egger (*p* = 0.05), there was no evidence of publication bias, however there is asymmetry in the funnel plot (Figure 7). From the sensitivity analysis, the country-income of the studies did not significantly (*p* > 0.05) affect child crystallised intelligence (Supplementary Figure S10). 

### 3.13. Outcome 7: Visual Processing

Three RCTs [45,53,55] measured the effect of a nutritional intervention on visual processing. There was no significant effect of choline supplementation (*n* = 1 trial) [45], multiple micronutrient supplementation (*n* = 1 trial) [53] or zinc supplementation (*n* = 1 trial) [55] on visual processing composite scores. 

### 3.14. Outcome 8: Attention

Four RCTs [47,66,74,76] measured the effect of a nutritional intervention on attention. There was no significant effect of multiple or LCPUFA supplementation (*n* = 2 trials) [47,74] on attention. Vuori et al. [76] reported that multiple micronutrient supplementation during pregnancy did positively affect attention in infants at 15 days postnatally. 

### 3.15. Outcome 9: Problem Solving

Two RCTs [59,64] measured the effect of a nutritional intervention on the problem solving composite score. There was no significant effect of multiple micronutrient intervention (*n* = 1 trial) [64] or LCPUFA intervention (*n* = 1 trial) [59] on problem composite scores. 

## 4. Discussion

This systematic review and meta-analysis indicates that nutritional interventions during pregnancy do not significantly impact on child cognitive outcomes. There is some evidence to suggest that LCPUFA supplementation may marginally improve child crystallised intelligence, however the effect was not statistically significant and study heterogeneity among the trials was significant. Although a wide scope of literature was assessed and a rigorous selection methodology was applied, the data included in this systematic review were affected by significant methodological limitations. From the included studies, 79% were exclusively nutrient supplementation trials, the effect of food-based interventions or nutrition counselling alone during pregnancy on child cognition was not well explored. Many studies were small, and attrition rates were high due to the length of follow-up required. Child cognition was a secondary outcome of interest in many studies, even with meta-analysis our findings are likely to be underpowered. This systematic was unable to perform a meta-analysis in specific age ranges due to the lack of data available for children older than 35 months, especially in low and middle income countries. Therefore, it is important that future trials include a longer child follow-up, to explore any possible delayed effects of maternal nutritional interventions on child cognition. 

### 4.1. Quality of Included Studies

Many included studies were considered to be of high quality. While, the Quality Checklist Criteria [36] is designed to assess the methodological quality and reporting within an article, it does not assess the appropriateness of the methodology in addressing the research question. This is a concern considering that many of included RCTs are likely to be at risk of type 2 error, due to the use of secondary data analysis and not powered to detect differences in cognitive outcome as a primary outcome variable. In addition, many post-hoc studies excluded participants after randomisation and in some cases multiple variable adjustments were required to address the significant characteristic differences between the groups, suggesting the assumptions of randomisation had been breached. Most post-hoc studies did not perform intention-to-treat analysis to evaluate the randomisation of the study groups. This approach is likely to introduce bias and systematic error that will reduce the power of the study. Therefore, it is important that future evidence is derived from adequately powered primary studies to reduce the risk of a type 2 error and systematic bias in post-hoc studies and determine whether there are any effects of nutritional interventions during pregnancy on child cognition. 

### 4.2. Measuring Child Cognition

There are many methodological challenges when measuring child cognition using psychological assessment tests. The cognitive performance of infants and children will be affected by their mood, motivation, amount of sleep and personal effort [138]. Environmental factors, including who conducts the cognition tests, who else is present during testing and where the tests are held can also influence a child’s performance ability [138]. In addition, most cognition tests were devised in developed countries, using stimuli relevant to their culture, therefore this stimuli is likely to be unfamiliar or unacceptable to children from developing countries. 

Many studies in this review used global development assessments (e.g., Bayley’s Scales of Infant Development) to determine the children’s overall cognitive ability. However, broad measures of cognitive function may only detect highly significant nutritional effects, especially if the study is underpowered [138]. Specific cognitive skills (e.g., attention, memory) that contribute to overall function are regulated by distinct neural mechanisms, that may be differentially affected by nutritional manipulations [139]. Therefore, Cognitive Assessment Tests that analyse specific skills, may be more sensitive to detect subtle effects of nutritional interventions on cognition. For this reason, future trials may be improved by the use of assessment tests that measure specific cognitive skills, as well as global measures of cognition. The choice of specific cognition test should be based on the hypothesised neural mechanisms that are expected to be altered by nutrients. 

To determine cognitive function later in childhood and adolescence, future studies should consider linking and analysing school performance data. Pilot data should be used to determine the appropriateness of the sample size and ensure that the study is adequately powered. This approach also has the advantage of the outcome assessors being blinded to the randomisation. 

### 4.3. Nutrition Intervention Design

Due to methodological and ethical challenges, most trials analysed the effects of nutrient supplementation during pregnancy on child cognition rather than using food-based interventions. This may reflect the perception that obtaining adherence to food-based interventions is a challenge and therefore nutrient supplementation is a more feasible strategy/study design. However, performing a meta-analysis for the supplementation trials proved to be difficult because many studies used a supplemented control group rather than an unsupplemented placebo group. Consequently, only the effect of the additional nutrient supplemented in the intervention group compared to the control group could be accessed. In Australia, evidence-based guidelines recommend the use of folic acid and iodine supplementation during pregnancy, therefore it is unethical to use an unsupplemented control group within this population. Li et al. [60] and Caulfield et al. [57] attempted to address this dilemma by providing the same dosage of folic acid across all study groups while women in the intervention groups received a multiple micronutrient supplement (including folic acid) or folic acid with iron. In addition, 88% of the studies conducted in high-income countries provided a single nutrient supplement to women during pregnancy. Since the effect of a single nutrient supplement on child cognition is likely to be modest, especially in a population that is well-nourished, this is likely to explain why a significant summary effect was not found. While, 88% of middle and low-income country studies, provided multiple micronutrient supplementation in a population that is likely to be undernourished, but no significant effect on child cognition was found. However, since child cognition assessment test were validated in a western population, this may have resulted in summary effect that was underestimated. 

By convention, many randomised control trials analyse the effect of nutrition on cognition by distilling complex dietary patterns into a single agent using a nutrient supplement. A single nutrient supplement is However, the bioavailability and efficacy of nutrient supplements can differ compared to nutrients contained in food [140]. Nutrient bioavailability is affected by the specific nutrient, absorptive capacity of intestinal mucosa, physiological states (e.g., pregnancy) and nutrient-nutrient interactions [140]. For example, the bioavailability of inorganic iron is enhanced by an interaction with vitamin C and increasing the bioavailability of iron can decrease magnesium and calcium absorption [140]. Huang et al. [141] reported that the bioavailability of natural vitamin was approximately 50 percent greater than synthetic vitamin E (α-tocopherol) in adults. In addition, synthetic vitamin E supplementation was also associated with a significant reduction in the bioavailability of other forms of vitamin E [141]. This trial highlights the limitations of analysing the effects of nutrition via a single agent without considering the biochemical pathways in which nutrients interact that is likely to be important for cognitive development [142,143,144]. Given the limited evidence available, there is a clear need for trials that are designed from a sound biologically plausible basis. 

### 4.4. Public Health Implications

Based on the meta-analyses presented, there is a lack of robust evidence on which to base recommendations for maternal diet or for the use of nutrient supplements as a strategy to enhance cognition in children. Given that the synergistic effect of micronutrients in metabolic pathways is important for enhancing cognitive development of children [145], it is important that future trials focus on food-based interventions that aim to improve the overall diet quality of pregnant women. In developing countries, pregnant women are more likely to experience protein-energy malnutrition, as well as multiple micronutrient deficiencies in comparison to high-income countries [146]. Therefore, further evidence is required to determine whether protein and energy as well as micronutrients interventions are effective for improving child cognition in the long-term in developing countries. 

## 5. Conclusions

In conclusion, this systematic review suggests that the infant and child cognitive outcomes were not significantly improved by nutritional interventions (dietary counselling, education, nutrition supplementation, fortified foods and/or whole foods). However, the possible association between LCPUFA supplementation and child crystallised intelligence warrants further investigation in high quality RCTs powered to detect difference in cognitive variables as the primary outcome. Further evidence from primary research is required to test whether maternal nutritional interventions during pregnancy, in particular food-based interventions, are beneficial for improving child cognition. This review emphasises the need for well-designed pilot studies to inform adequately powered RCTs and data linkage studies. This evidence is important because gains in child cognition are likely to have extended reach to other areas of public health and carry economic benefit. 

## Figures and Tables

**Table 1:** Inclusion and exclusion criteria for selecting studies.

Criteria	Study Design	Population	Intervention	Outcome
Include	Randomised or pseudorandomised controlled trials of any date	Pregnant women of any age or ethnicity	Dietary intervention/s, including dietary counselling and education as well as food/s, fortified foods or nutrient supplement/s	Measures cognitive outcomes of infants and children using cognitive assessment tests
		Singleton pregnancies	Dietary intervention/s provided during pregnancy (exclusively) or intervention/s commencing during gestation and continued during lactation	Cognition is measured after birth in children <10 years of age
Exclude	All other designs including animal studies	Women that are not pregnant	Dietary intervention/s are not provided to pregnant women	Cognitive outcomes are not measured in children after pregnancy
		Multiple births for the primary population	Dietary intervention/s that commence after pregnancy	

**Table 2:** Overview of the characteristics of the randomised controlled trials (RCTs).

Source, Year, Country, OECD	Infant Sample (*n*)	Intervention Group/s	Control Group/s	Intervention Duration	Cognitive Testing Age	Cognitive Assessment Tests	Main Findings
**Choline Intervention vs. Control**							
Cheatham et al. [45] 2012, US, 1	99	Supplement: Vitamins: 750 mg/day choline	Supplement: corn oil capsules	18 weekends gestation to 3 months postnatally	10 and 12 month	Visuospatial Memory Delayed Response Task [78,79,80], Imitation paradigms [81], CDI [82] & MSEL [83]	There were no significant differences between the intervention and control groups for global development, language, short-term visuospatial memory, or long-term episodic memory test scores (*p* > 0.05) at 10 and 12 months
**Iodine Intervention vs. Control**							
Santiago et al. [54], Spain, 1	102	Supplement: Mineral: I1: 200 µg/day iodine I2: 300 µg/day iodine	Fortified food: used iodised salt	Before 10 weekends gestation to delivery	6 to 18 month	BSID-III [34]	There were no significant differences between the groups for MDI and PDI scores (*p* > 0.05) at 6 to 18 months
Zhou et al. [70], Australia, 1	53	Supplement: Mineral: 150 µg/day iodine	No supplement	Less than 20 weekends gestation to delivery	18 month	BSID-III [34]	There was no significant difference between the intervention compared to the control group for child cognition (*p* = 0.42), language (*p* = 0.83) and motor skills (*p* = 0.61) at 18 months of age
Zhou et al. [65], Australia, 1	302	Supplement: Mineral: 20 mg/day Fe	No supplement	20 weekends gestation to delivery	48 months	SB4 [84] & SDQ [85]	There were no significant differences between groups for child cognition and behaviour test scores (*p* > 0.05) at 48 months
**Iron and Folic Acid or Multiple Micronutrient Intervention vs. Control**							
Chang et al. [73], China, 3	850	Supplement: Vitamins: I1: 400 µg/day FA I2: 400 µg/day FA, 800 µg/day vit A, 1.4 mg/day vit B1, 1.4 mg/day vit B2, 18 mg/day B3, 1.9 mg/day vit B6, 2.6 µg/day vit B12, 70 µg/day vit C, 5 µg/day vit D, 10 mg/day vit E Minerals: I1: 60 mg/day Fe I2: 2 mg/day Cu, 150 µg/day iodine, 30 mg/day Fe, 65 µg/day Se, 15 mg/day Zn	Supplement: Vitamin: 400 µg/day FA	Less than 28 weekends gestation to delivery	3,6,12,18 and 24 months	BSID-II (Chinese translation) [86,87]	The prenatal IDA intervention groups had a lower MDI (*p* = 0.046 in folic acid; *p* = 0.034 in multiple micronutrient supplements) compared to the prenatal-non-IDA group. There was no significant difference in MDI scores in the prenatal-IDA group and prenatal-non-IDA group that received iron and folic acid supplements (*p* = 0.641)
Li et al. [60], China, 2	1305	Supplement: Vitamins: I1: 800 µg/day vit A, 1.4 mg/day vit B1, 1.4 mg/day B2, 18 mg/day vit B3, 1.9 mg/day vit B6, 2.6 mg/day vit B12, 70 mg/day vit C, 10 µg/day vit D, 10 mg/day vit E, 400 µg/day FA I2: 400 µg/day FA Minerals: I1: 2 mg/day Cu, 30 mg/day Fe, 150 µg/day iodine, 65 µg/day Se I2: 30 mg/day Fe	Supplement Vitamins: 400 µg/day FA	27 weekends gestation to delivery	3, 6 and 12 months	BSID-II [86]	Positive effects were demonstrated from the multiple micronutrient supplement on child MDI scores at 12 months (*p* = 0.01)
Hanieh et al. [48], Vietnam, 3	1168	Supplement taken twice weekly Vitamins: I1: 1500 µg/day FA I2: 1600 µg/day vit A, 2.8 mg/day vit B1, 2.8 mg/day vit B2, 36 mg/day vit B3, 3.8 mg/day vit B6, 2.8 mg/day vit B12, 140 mg/day vit C, 10 mg/day vit D, 20 mg/day vit E, 1500 µg/day FA Minerals: I1: 60 mg/day Fe I2: 4 mg/day Cu, 60 mg/day Fe, 300 µg/day iodine, 130 µg/day Se	Supplement taken daily Vitamin: 400 µg/day FA Mineral: 60 mg/day Fe	Less than 16 weekends gestation to 3 months postnatally	6 months	BSID-III [34]	A positive effect was demonstrated from the twice weekly iron and folate acid supplement groups on child cognitive development scores (MD 1.89; 95% CI 0.23 to 3.56) at 6 months
**LCPUFA s Intervention vs. Control**							
Brei et al. [72], Germany, 1	130	Supplement: fish oil capsules LCPUFAs: 1020 mg/day DHA, 180 mg/day EPA Vitamin: 9 mg/day vit E Dietary counselling to lower ARA intake	Participants received information for eating a healthy diet during pregnancy	15 weekends gestation to 4 months postnatally	60 months	Child Development Inventory (German translation) [88,89], MM [90,91]	There were no differences between the maternal fish oil supplemented group compared to the control group on child CDI scores and MM at 4 and 5 years of age (*p* > 0.05)
Dunstan et al. [46], Australia, 1	72	Supplement: fish oil capsules LCPUFAs: 2.2 g/day DHA, 1.1 g/day EPA Vitamins: 3–4 mg/day vit E	Supplement: olive oil capsules LCPUFA: 2.7 g/day oleic acid Vitamins: 3–4 mg/day vit E	20 weekends gestation to delivery	18 months	GMDS 0-2 [92], PPVT-III [93] & CBCL [94]	A positive effect was demonstrated from the maternal fish oil intervention on eye and hand coordination (*p* = 0.021) in children at 18 months
Gould et al. [47], Australia, 1	185	Intervention: marine oil capsules LCPUFAs: 0.8 g/day DHA, 0.1 g/day EPA	Supplement: capsule with a blend of vegetable oils	18–21 weekends gestation to delivery	27 months	Single and multiple object task [95,96] & A-not-B task [97]	There were no significant differences between the intervention and control groups for child attention, working memory and inhibitory control development test scores (*p* > 0.05)
Helland et al. [49], Norway, 1	262	Supplement: cod liver oil LCPUFAs: 75 mg/day ALA, 27.5 mg/day ARA, 1.18 g/day DHA, 0.8 g/day EPA, 160 mg/day LA, Vitamins: 117 µg/day vit A, 1 µg/day vit D, 1.4 mg/day vit E	Supplement: corn oil LCPUFAs: 92 mg/day ALA, 8.3 mg/day DHA, 4.7 g/day LA Vitamins: 117 µg/day vit A, 1 µg/day vit D, 1.4 mg/day vit E	17–19 weekends gestation to 3 months postnatally	27 and 39 weekends	FTII-II [98]	There were no significant differences between the intervention and control groups for cognitive development test scores (*p* > 0.05) at 27 and 39 months
Helland et al. [51], Norway, 1	90	Supplement: cod liver oil LCPUFAs: 75 mg/day ALA, 27.5 mg/day ARA, 1.18 g/day DHA, 0.8 g/day EPA, 160 mg/day LA, Vitamins: 117 µg/day vit A, 1 µg/day vit D, 1.4 mg/day vit E	Supplement: corn oil LCPUFAs: 92 mg/day ALA, 8.3 mg/day DHA, 4.7 g/day LAVitamins: 117 µg/day vit A, 1 µg/day vit D, 1.4 mg/day vit E	1–19 weekends gestation to 3 months postnatally	48 months	KABC-II [99]	Apositive effect was demonstrated from the cod liver oil supplement on child mental processing composite score (*p* = 0.049) at 48 months
Helland et al. [50], Norway, 1	143	Supplement: cod liver oil LCPUFAs: 75 mg/day ALA, 27.5 mg/day ARA, 1.18 g/day DHA, 0.8 g/day EPA, 160 mg/day LA, Vitamins: 117 µg/day vit A, 1 µg/day vit D, 1.4 mg/day vit E	Supplement: corn oil LCPUFAs: 92 mg/day ALA, 8.3 mg/day DHA, 4.7 g/day LA Vitamins: 117 µg/day vit A, 1 µg/day vit D, 1.4 mg/day vit E	1–19 weekends gestation to 3 months postnatally	84 months	KABC-II [99]	No significant differences between the intervention and control groups for cognitive development test scores (*p* > 0.05) at 84 months was found
Hurtado et al. [68], Spain, 1	61	Supplement: fish oil drink per 100 mL Energy: 145 kJ Macronutrients: 3.7 g prot., 6.7 g carb., 1.8 g total fat LCPUFAs: 80 mg DHA, 18 mg EPA Vitamins: 0.4 µg vit B12, 9 mg vit C, 0.75 µg vit D, 1.5 mg vit E, 80 µg FA Minerals: 23 µg iodine, 2.2 mg Fe	Supplement drink per 100 mL Energy: 145 kJ Macronutrients: 3.7 g prot., 6.7 g carb., 1.8 g total fat Vitamins: 0.4 µg vit B12, 9 mg vit C, 0.75 µg vit D, 1.5 mg vit E, 80 µg FA Minerals: 23 µg iodine, 2.2 mg Fe	28 weekends gestation to 4 months postnatally	12 months	BSID-II [86]	There were no differences between the maternal fish oil supplemented group compared to the control group on child MDI and PDI composite scores at 12 months of age
Judge et al. [59], US, 1	29	Muesli bar with fish oil. Per bar; Energy: 386 kJ Macronutrients: 18 g carb., 0.3 g fibre, 1.3 g prot., 1.7 g total fat, 300 mg DHA, 38 mg EPA	Muesli bar without fish oil. Per bar; Energy: 386 kJ Macronutrient: 18 g carb., 0.3 g fibre, 1.3 g prot., 1.7 g total fat	24 weekends gestation until delivery	9 months	2-step problem-solving test [100,101,102,103] & FTII-II [98]	Positive effects were demonstrated from the fish oil intervention group on the problem-solving task (*p* = 0.017), total intentional score (*p* = 0.011) and the number of intentional solution on both cloth (*p* = 0.008) and cover steps (*p* = 0.004) at 9 months
Makrides et al. [52], Australia, 1	726	Supplement: fish oil capsules LCPUFAs: 800 mg/day DHA, 100 mg/day EPA	Supplement: Capsules with vegetable oil blend	21 weekends gestation to delivery	18 months	BSID-III [34]	There were no significant differences between the groups for cognitive and language test scores (*p* > 0.05)
Mulder et al. [62], Canada ^4^, 1	216	Supplement: algal oil capsules LCPUFAs: 400 mg/day DHA	Supplement: soybean and corn oil capsules	16 weekends gestation to delivery	9 and 18 months	CDI [82] & BSID-III [34]	Negative effects were demonstrated from the maternal algal oil supplement for CDI language (*p* < 0.05) at 14 months and 18 months and BSID-III language scores at 18 months (*p* < 0.05)
Ramakrishnan et al. [74], Mexico, 2	797	Supplement: algal oil capsules LCPUFAs: 400 mg/day DHA	Supplement: corn and soy oil capsules	18–22 weekends Gestation to delivery	60 months	MSCA (Spanish translation) [104], (BASC-2) [105], and (K-CPT) [106]	There were no significant differences between the intervention group compared to the control group for MSCA scores (*p* > 0.05), BASC-2 or scores at 5 years. of age. The intervention did significantly affect K-CPT omissions scores (*p* = 0.01) compared to the placebo group
Tofail et al. [63], Bangladesh, 4	249	Supplement: fish oil capsules LCPUFAs: 1.2 g/day DHA, 1.8 g/day EPA	Supplement: soy oil capsules LCPUFAs: 2.3 g/day LA	27 weekends gestation to delivery	10 months	BSID-II [86] & Wolke’s Behaviour Rating Scale [107]	There were no significant differences between the intervention and control groups for cognitive or behaviour development test scores (*p* > 0.05) at 10 months
Van Goor et al. [56], Netherlands, 1	114	Fish oil capsules I1: 220 mg/day DHA, 34 mg/day EPA, 15 mg/day ARA, 274 mg/day LA, 32 mg/day ALA I2: 220 mg/day DHA, 36 mg/day EPA, 220 mg/day ARA, 46 mg/day LA, 7 mg/day ALA	Supplement: soy bean oil capsules	14–20 weekends gestation to 3 months postnatally	18 months	BSID-II [86]	There were no significant differences between the control and intervention groups for cognitive development test scores (*p* > 0.05) at 18 months
**LCPUFA and Folic Acid Intervention vs. Control**							
Catena et al. [66], Germany, Spain & Hungary, 1	136	Dairy drink per sachet: I1: 500 mg/day DHA, 150 mg/day EPA I2: 400 µg/day FA I3: 500 mg/day DHA, 150 mg/day EPA, 400 µg/day FA	Dairy drink with vitamins and minerals in accordance to European dietary guidelines for pregnancy	Less than 20 weekends gestation to delivery	102 months	Attention Network Test (child version) [108,109]	Children born to mothers supplemented with folic acid alone solved the response conflict more quickly than the control group and the fish oiand folic acid group (all *p* < 0.05)
**Multiple Micronutrient Intervention vs. Control**							
Christian et al. [71], Nepal, 4	676	Supplement: Vitamins: I1: 1000 µg/day vit A, 400 µg/day FA I2: 1000 µg/day vit A, 400 µg/day FA I3: 1000 µg/day vit A, 400 µg/day FA I4: 1000 µg/day vit A, 400 µg/day FA, 1.6 mg/day vit B1, 1.8 mg/day vit B2, 20 mg/day vit B3, 2.2 µg/day vit B6, 2.6 µg/day vit B12, 100 mg/day vit C, 10 µg/day vit D, 10 mg/day vit E, 65 µg/day vit K Minerals: I2: 60 mg/day Fe I3: 60 mg/day Fe, 30 mg/day Zn I4: 2 mg/day Cu, 60 mg/day Fe, 100 mg/day Mg, 30 mg/day Zn	Supplement: Vitamin: 1000 µg/day vit A	Pregnancy to 3 months postnatally	84–108 months	UNIT [110], Go/no-go [111], the Stroop test [112], backward digit span [113], MABC [114], finger-tapping test [115]	The difference across all outcomes (UNIT, executive function and motor functioning) was significant in children whose mother received iron and folic acid supplementation (*p* = 0.001) compared to the control group but not for the other intervention groups (*p* > 0.05)
Christian et al. [67], Bangladeshi, 4	734	Supplement: Vitamins: 770 µg/day vit A, 1.4 mg/day vit B1, 1.4 mg/day vit B2, 18 mg/day vit B3, 1.9 mg/day vit B6, 2.6 µg/day vit B12, 85 mg/day vit C, 5 µg/day vit D, 15 mg/day vit E, 600 µg/day FA Minerals: 1000 µg/day Cu,220 µg/day iodine, 27 mg/day Fe, 60 µg Se, 12 mg/day Zn	Supplement: Vitamin: 600 µg/day FA Mineral: 27 mg/day Fe	Pregnancy to 3 months postnatally	24 months	BSID-III [34]	There were no differences between the intervention and control group on child composite scores for cognition (*p* = 0.52), language (*p* = 0.97), and motor performance (*p* = 0.22) at 24 months of age
Joos et al. [116], Taiwan, 2	198	Supplement: 1680 kJ, 50 g /day carb., 0.55 g/day fibre, 20 g/day prot., 13.3 g/day total fatVitamins: 750 µg/day vit A, 0.8 mg/day vit B1, 0.9 mg/day vit B2, 10 mg/day vit B3, 0.08 mg/day vit B6, 1 mg/day vit B12, 37.5 mg/day vit C, 5 µg/day vit D, 3.35 mg/day vit E Minerals: 0.5 mg/day Ca, 0.5 mg/day Cu, 6 mg/day Fe, 1 mg/day Mn, 0.9 mg/day K, 0.2 mg/day Na, 0.4 mg/day	No supplement	Conception to lactation of 2nd child	8 months	BSID-I [117]	A positive effect was demonstrated from the multiple micronutrient supplement on PDI (mean ± SD: 3.80 ± 1.90 compared to 3.31 ± 1.71) at 8 months
Prado et al. [53], Indonesia, 3	487	Supplement Vitamins: 800 µg/day vit A, 1.4 mg/day vit B1, 1.4 mg/day vit B2, 18 mg/day vit B3, 1.9 mg/day vit B6, 2.6 mg/day vit B12, 70 mg/day vit C, 5 µg/day vit D, 100 mg vit E, 400 µg/day FA Minerals: 2 µg/day Cu, 30 mg/day Fe, 150 µg/day iodine, 65 µg/day Se	Supplement: Vitamins: 400 µg/day FA Minerals: 30 mg/day Fe	Pregnancy to 3 months postnatally	42 months	BSID-II [86], ASQ [118], BPVS-II [119], BAS-2 [120], CDI [82], NEPSY [121], Snack Delay Test [122,123], Windows Test [124] & Socioemotional Development Scale [53].	No significant differences between the intervention and control groups for child motor, language, visual attention/spatial, executive, and socio-emotional development test scores (*p* > 0.05) at 42 months was found
Tofail et al. [64], Bangladesh, 4	2853	Food & supplements Energy: 2512 kJ. Vitamins: I1: 400 µg folate I2: 1.4 mg/day vit B1, 1.4 mg/day vit B2, 18 mg/day vit B3, 2.6 mg/day vit B6, 1.9 mg/day vit B12, 70 mg/day vit C, 5 µg/day vit D, 10 mg/day vit E, 400 µg/day FA Minerals: I1: 30 mg/day Fe I2: 2 mg/day Cu, 60 mg/day Fe, 150 µg/day iodine, 65 µg/day, Se 15 mg/day Zn	Food & supplements 2512 kJ. Vitamins: I1: 400 µg folate I2: 1.4 mg/day vit B1, 1.4 mg/day vit B2, 18 mg/day vit B3, 2.6 mg/day vit B6, 1.9 mg/day vit B12, 70 mg/day vit C, 5 µg/day vit D, 10 mg/day vit E, 400 µg/day FA Minerals: I1: 30 mg/day Fe I2: 2 mg/day Cu, 60 mg/day Fe, 150 µg/day iodine, 65 µg/day, Se 15 mg/day Zn	17 weekends gestation to delivery	7 months	Two step problem-solving tests [100,101,102,103], BSID-II [86] & Wolke’s Behaviour Rating Scale [107]	There were no significant differences between the intervention and control groups for child problem solving ability, cognitive or behaviour development test scores (*p* > 0.05) at 7 months
Vuori et al. [76], Colombia, 2	244	Food & supplements: Energy: 3595 kJ Prot: 38.4 g/day Vitamin: 1807 µg/day vit A Mineral: 18 mg/day Fe	No supplement	27 weekends gestation and continued during lactation	15 days	Checkboard activity [76]	Positive effects were demonstrated from food supplements on infants that achieved habituation (70%) in comparison to the control group (56%) *p* < 0.05. In addition the supplemented infants moved less during testing (mean = 11.80, SD = 6.85) in comparison to the supplemented group (mean = 10.19, SD = 5.53)
Waber et al. [125], Colombia, 2	304	Food, supplements & nutrition education 3595 kJ energy, 38.4 g prot., 25.8 g total fat. Vitamins: 1807 µg vit A, 5.4 mg vit B1, 6.6 mg vit B2, 31.6 mg vit B3, 104.6 mg vit C Mineral: 1146 mg Ca	No supplement	27 weekends gestation to 6 months postnatally	4, 6, 12, 18, 24 and 36 months	GMDS 0–2 [92] & Escalona & Corman’s Albert Einstein Scales of Sensorimotor Development [126]	Positive effects were demonstrated from food supplements on subtests including personal social (*p* = 0.006), speech and language (*p* = 0.019), eye and hand coordination (*p* = 0.008), as well as general quotient (*p* = 0.003)
**Multiple Micronutrient or Vitamin A Intervention vs. Control**							
McGrath et al. [61], Tanzania, 4	327	Supplement: Vitamins: I1: 1500 µg/day vit A, 30 mg/day beta-carotene, 20 mg/day vit B1, 20 mg/day vit B2, 100 mg/day vit B3, 25 mg/day vit B6, 50 mg/day vit B12, 500 mg/day vit C, 30 mg/day vit E, 800 µg/day FA I2: 1500 µg/day vit A, 30 mg/day beta-carotene, 20 mg/day	Supplement: Vitamins: C1: 20 mg/day vit B1, 20 mg/day vit B2, 100 mg/day vit B3, 25 mg/day vit B6, 50 mg/day vit B12, 500 mg/day vit C, 30 mg/day vit E, 800 µg/day FA C2: No supplements	12–27 weekends gestation to 18 months in postnatal period	6, 12 and 18 months	BSID-II [86]	Positive effects were demonstrated from the multiple micronutrient supplement by increasing PDI score by 2.6 points (95% CI: 0.1–5.1) over 6 to 18 months Vitamin A supplementation was also associated with 2.8 point increase (95% CI: 0.4-5.2) in PDI score at 6 months
**Vitamin A, Folic Acid and Iron Intervention vs. Control**							
Schmidt et al. [69], Indonesia, 4	188	Supplement: Vitamins: 4800 µg/day vit A, 500 µg/day FA Mineral: 120 mg/day Fe	Supplement: Vitamin: 500 µg/day FA Mineral: 120 mg/day Fe	16–20 weekends gestation to delivery	6 and 12 months	BSID-I [117]	There were no significant effects of vitamin A supplementation on MDI and PDI scores at 6 or 12 months of age (*p* > 0.05). There was no significant difference between weekly compared to daily iron supplementation on child MDI and PDI scores (*p* > 0.05)
**Vitamin B12 Intervention vs. Control**							
Srinivasan et al. [75], India, 3	178	Supplement: Vitamin: 50 µg/day vit B12	No supplement	Less than 14 weekends gestation to 6 weekends postnatally	9 months	BSID-III [34]	There were no significant effects of maternal B12 supplementation on child MDI and PDI scores at 9 months (*p* > 0.05)
**Zinc Intervention vs. Control**							
Hamadani et al. [58], Bangladesh, 4	168	Supplement: Vitamin: 30 mg/day Zn	No supplement	Pregnancy to delivery	13 months	BSID-II [86] & Wolke’s Behaviour Rating Scale [107]	Negative effects were demonstrated from maternal zinc supplementation on child MDI (*p* = 0.04) and psychomotor development index (PDI) (*p* = 0.04) at 13 months
Tamura et al. [55], US, 1	347	Supplement: Mineral: 25 mg/day Zn	No supplement	19 weekends gestation to delivery	60 months	DAS [127], VSM [128], ASM [128], Knox Cube [129], PDMS-2 [130] & Grooved Pegboard [131]	There were no significant differences between the intervention and control groups for cognitive, memory or motor development test scores (*p* > 0.05) at 60 months
**Zinc, Folic Acid and Iron vs. Control**							
Caulfield et al. [57], Peru, 2	184	Supplement: Vitamins: 250 µg/day FA Minerals: 25 mg/day Zn, 60 mg/day Fe	Supplement: Vitamins: 250 µg/day FA Minerals: 60 mg/day Fe	10–14 weekends gestation to delivery	54 months	WPPSI-III [113], bear story [132,133], the counting game [57], draw a person [134], the friendship interview [135,136], Vinelands II [97] & PBQ [137]	There were no significant differences between the intervention and control groups for child cognitive, social or behavioural development test scores (*p* > 0.05) at 54 moths

ALA: alpha-linolenic acid; ARA: arachidonic acid; ASQ: Ages and Stages Questionnaire, second edition; ASM: Auditory Sequential Memory; BAS-II: British Ability Scales, second edition; BASC-2: Behavioural Assessment System for Children; BPVS: British Picture Vocabulary Scale, second edition; BSID-I: Bayley Scales of Infant Development, first edition; BSID-II: Bayley Scales of Infant Development, second edition; BSID-III: Bayley Scales of Infant Development, third edition; C1: control 1; C2: control 2, Ca: calcium; CBCL: Child Behaviour Checklist; Carb: carbohydrates; CDI: MacArthur-Bates Communicative Development Inventories; CI; confidence interval; CL: confidence level; Cu: copper; DAS: Differential Ability Scales, first edition; DHA: docosahexaenoic acid; EPA: eicosapentaenoic acid; FA: folic acid; Fe: iron; I1: intervention 1; FTII-II: Fagan Test of Infant Intelligence, second edition; I2: intervention 2; I3: intervention 3; I4: intervention 4; IDA: iron deficiency anaemia; GMDS 0–2: Griffiths Mental Development Scales for ages 0–2 years; K: potassium; KABC-II: Kaufman Assessment Battery for Children, second edition; K-CPT: Conners’ Kiddie Continuous Performance Test; LA: linoleic acid; LCPUFA: long chain polyunsaturated fatty acids; MABC: Movement Assessment Battery for Children; MD: mean difference; MDI: motor development index; MM: lower mirror movements; Mn: manganese; MSCA: McCarthy Scales of Children’s Abilities; MSEL: Mullen Scales of Early Learning; Na: sodium; NEPSY-II: A Developmental Neuropsychological Assessment, second edition; OR: odds ratio; P: phosphorus; PDI: psychomotor development index; PBQ: Preschool Behaviour Questionnaire; PDMS-2: Peabody Developmental Motor Scales, 2nd edition; PPVT-III: Peabody Picture Vocabulary Test, third edition; Prot: protein; SB4: The Stanford-Binet Intelligence Test; SD: standard deviation; DQ: Strength and Difficulties Questionnaire; Se: selenium; SE: standard error; UNIT: Universal Non-verbal Intelligence Test; Vineland II: Vineland’s Adaptive Behaviour Scales, second edition; VSM: Visual Sequential Memory; Vit: vitamin; WPPSI-III: Wechsler Preschool Primary Scale of Intelligence, third edition; Zn: zinc. OECD, the organisation for economic co-operation and development criteria: 1 = high income country, 2 = higher middle income country, 3 = lower middle income country & 4 = low income country.

**Table 3:** Meta-analyses for cognitive outcomes in evaluation of nutritional interventions during pregnancy.

Dietary Nutrient	Cognitive Outcome	Study Authors	Country (OECD)	Studies (*n*)	Children (*n*)	SMD (95% CI) ^†^	*p*-Value	*I*^2 ‡^
LCPUFA	Attention	Gould et al. [47] Ramakrishnan et al. [74]	Australia (1) Mexico (2)	2	955	−0.07 (−0.17 to 0.03)	0.19	0%
LCPUFA	Behaviour	Dunstan et al. [46] Makrides et al. [52] Brei et al. [72] Ramakrishnan et al. [74]	Australia (1) Australia (1) Germany (1) Mexico (2)	4	1725	−0.05 (−0.12 to 0.03)	0.25	0%
MMN	Motor skills	Li et al. [60] McGrath et al. [61] Prado et al. [53] Chang et al. [73] Christian [71] Christian [67]	China (2) Tanzania (4) Indonesia (3) China (3) Nepal (4) Bangladesh (4)	6	3572	0.02 (−0.04 to 0.17)	0.55	0%
LCPUFA	Motor skills	Dunstan et al. [46] Makrides et al. [52] Mulder et al. [62] Tofail et al. [63] Van Goor et al. [56] Brei et al. [72] Ramakrishnan et al. [74]	Australia (1) Australia (1) Canada (1) Bangladesh (4) Netherlands (1) Germany (1) Mexico (2)	7	2265	0.06 (−0.03 to 0.15)	0.22	8.9%
Zinc	Motor skills	Caulfield et al. [57] Hamadani et al. [58] Tamura et al. [55]	Peru (2) Bangladesh (4) United States (1)	3	985	−0.10 (−0.38 to 0.17)	0.49	72.5%
LCPUFA	Fluid intelligence	Brei et al. [72] Dunstan et al. [46] Ramakrishnan et al. [74]	Germany (1) Australia (1) Mexico (2)	3	999	0.05 (−0.08 to 0.18)	0.45	10.1%
MMN	Fluid intelligence	Christian et al. [71] Prado et al. [53]	Nepal (4) Indonesia (3)	2	755	0.07 (−0.20 to 0.33)	0.63	78.2%
Zinc	Fluid intelligence	Caulifield et al. [57] Tamura et al. [55]	Peru (2) United States (1)	2	539	−0.10 (−0.25 to 0.06)	0.23	0%
MMN	Global cognition	Joos et al. [116] Li et al. [60] McGrath et al. [61] Waber et al. [125] Chang et al. [73] Christian et al. [67]	Taiwan (2) China (2) Tanzania (4) Colombia (2) China (3) Bangladesh (4)	6	3126	0.09 (−0.02 to 0.19)	0.11	57.2%
LCPUFA	Global cognition	Dunstan et al. [46] Helland et al. [49] Helland et al. [51] Judge et al. [59] Makrides et al. [52], Mulder et al. [62] Tofail et al. [63] Van Goor et al. [56] Brei et al. [72] Ramakrishnan et al. [74]	Australia (1) Norway (1) Norway (1) United States (1) Australia (1) Canada (1) Bangladesh (4) Netherlands (1) Germany (1) Mexico (2)	10	2632	0.03 (−0.07 to 0.13)	0.55	21.3%
Zinc	Crystallised intelligence	Caulfield et al. [57] Tamura et al. [55]	Peru (2) United States (1)	2	539	−0.04 (−0.20 to 0.12)	0.61	0%
LCPUFA	Crystallised intelligence	Dunstan et al. [46] Makrides et al. [52] Mulder et al. [62] Brie et al. [72] Ramakrishnan et al. [74]	Australia (1) Australia (1) Canada (1) Germany (1) Mexico (2)	5	1941	0.25 (−0.04 to 0.53)	0.09	87.8%
MMN	Crystallised intelligence	Christian et al. [67] Prado et al. [53]	Bangladesh (4) Indonesia (3)	2	1207	0.01 (−0.11 to 0.12)	0.91	0%

CI: confidence interval; LCPUFA: long chain polyunsaturated fatty acids; MMN: multiple micronutrient; SMD: standardised mean difference; OECD: the organisation for economic co-operation and development criteria; 1 = high income country, 2 = higher middle income country, 3 = lower middle income country & 4 = low income country; ^†^ the main measure of effect was SMD (also known as Cohens d). The SMD was determined by taking the difference between the mean of the intervention group compared to the control group, and dividing the pooled standard deviation for the outcome across the whole trial. A random effects model using the method DerSimonian & Laird [43] was applied to the data; ^‡^ the *I*^2^ statistic is the percentage of observed total variation across studies that is due to heterogeneity rather than chance. It is calculated using the following formula: *I*^2^ = 100% × (*Q* − *df*)/*Q*, where *Q* is Cochran’s heterogeneity and *df* is the degrees of freedom [44].

## Supplementary Materials

The following are available online at www.mdpi.com/2072-6643/9/11/1265/s1, Table S1: Search strategy used for systematic review; Table S2: Computing a combined effect across outcomes; Table S3: Summary of quality assessment; Table S4: Summary of the nutritional interventions for included studies; Table S5: Cognitive Assessment Tests; Figure S1: Review protocol; Figure S2: Funnel plot: behavior; Figure S3: Sensitivity analysis: behavior; Figure S4: Funnel plot: motor skills; Figure S5: Sensitivity analysis: motor skills; Figure S6: Funnel plot: fluid intelligence; Figure S7: Sensitivity analysis: fluid intelligence; Figure S8: Funnel plot: global cognition; Figure S9: Sensitivity analysis: global cognition; Figure S10: Sensitivity analysis: crystallised intelligence.

## Abbreviations

CHC	Cattell–Horn–Carroll
CI	confidence interval
DHA	docosahexaenoic acid
HIV-1	human immunodeficiency virus type 1
LCPUFA	long chain polyunsaturated fatty acids
OECD	Organisation for Economic Co-operation and Development
PRISMA	Preferred Reporting Items for Systematic Review Meta-Analyses
PRISMA	randomised controlled trials
SMD	standardised mean difference
