# Supplementary material for: Effects of Nutritional Interventions during Pregnancy on Infant and Child Cognitive Outcomes: A Systematic Review and Meta-Analysis
Source: Nutrients. 2017 Nov 20;9(11):1265. doi: 10.3390/nu9111265 (PMC5707737; doi:10.3390/nu9111265)
Supplement: Supplementary file 1 [file nutrients-09-01265-s001.pdf]

Supplementary Table S1. (a) Search strategy used for systematic review

	**NUMBER OF SEARCH RESULTS**			
**DATABASE**	Medline	EMBASE	PsycINFO	Maternity & Infant Care
**DATE SEARCHED**	03/08/17	03/08/17	03/08/17	03/08/117
1. pregnan*.mp.	932948	957161	43435	100664
2. Cogniti*.mp.	363067	535861	500106	3234
3. intelligen*.mp.	79604	101702	127584	951
4. neurodevelopment.mp.	6710	9709	2774	787
5. memory.mp.	237631	327420	202937	646
6. attention.mp.	378627	494577	241148	4410
7. language.mp.	153663	191869	202574	2955
8. infant.mp.	1115605	738338	51953	65811
9. child.mp.	1942198	2203481	305023	25162
10. randomi?ed control* trial.mp.	497544	613546	16943	4081
11. clinical trial.mp.	673148	1373681	12163	1566
12. placebo.mp.	198177	405667	36426	2900
13. randomly.mp.	287183	362322	62967	4800
14. food*.mp.	525037	753733	78346	4798
15. nutrition.mp.	201434	285767	18607	6810
16. diet.mp.	386578	658464	21574	4109
17. dietary supplement*.mp.	54834	22780	2084	1071
18. nutrient supplement*.mp.	980	1283	64	65
19. 2 or 3 or 4 or 5 or 6 or 7	1023919	1372984	1009405	11028
20. 8 or 9	2457606	2511550	337291	79210
21. 10 or 11 or 12 or 13	1131989	1933554	115983	10913
22. 14 or 15 or 16 or 17 or 18	983470	1424308	103949	12592
23. 1 and 19 and 20 and 21 and 22	134	278	9	17
24. limit 23 to (English language and humans)	134	271	9	No limits

Supplementary Table S1. (b) Search strategy used for systematic review

	**NUMBER OF SEARCH RESULTS**				
**DATABASE**	**CINAHL**	**Web of Science**	**Scopus**	**Proquest**	**Pre-Medline**
**SEARCH DATE**	03/08/17	03/08/17	04/08/17	04/08/17	04/08/17
1. Pregnan*	178145	417674	1046879	22391	30707
2. Cognit* OR neurodevelopment	124551	533717	702608	26510	43383
3. Infant* OR child*	673543	1721434	3383507	112028	110508
4. Randomi?sed control* trial OR clinical trial	184567	551358	1981053	17302	17302
5. Food* OR nutrition OR supplement*	240816	1160592	1852193	110508	76965
1 AND 2 AND 3 AND 4 AND 5	20	58	304	15	4
LIMITS APPLIED	No limits	No limits	Excluded Book chapters Editorials Note Letter Summary Limited to English: 162	Limited to Peer reviewed & scholarly journals	No limits

Supplementary Table S2. Computing a combined effect across outcomes

Author	Year	Composite outcome	Mean intervention (sd)	Mean control (sd)	SMD	Combined SMD (se)
Gould	2014	Attention subtest score	3.5 (1.6)	3.8 (1.9)	-0.17 (0.16)	-0.04 (0.13)
Gould	2014	Attention subtest score	41.6 (11.8)	42 (12.4)	-0.03 (0.16)	-0.04 (0.13)
Gould	2014	Attention subtest score	238.8 (51.1)	246.6 (41)	-0.17 (0.16)	-0.04 (0.13)
Gould	2014	Attention subtest score	132.6 (55.3)	120 (63.4)	0.21 (0.16)	-0.04 (0.13)
Vuori	1987	Attention subtest score	10.19 (5.53)	11.8 (6.85)	-0.26 (0.13)	-0.26 (0.13)

Supplementary Table S3. Summary of the quality assessment for the included studies [1]

First author, year of publication (reference)	Brei, 2017	Catena, 2016	Caulfield, 2010	Cheatham, 2012	Chang, 2013	Christian, 2010	Christian, 2016	Dunstan, 2008	Gould, 2014	Hamandani, 2002	Hanieh, 2013	Helland, 2001	Helland, 2003	Helland, 2008	Hurtado, 2015	Joos, 1983	Judge, 2007	Li, 2009	Makrides, 2010	McGrath, 2006	Mulder, 2014	Prado, 2012	Ramakrishnan, 2016
**VALIDITY QUESTIONS**																							
1. Was the research question clearly stated?	Y	Y	Y	Y	Y	Y	Y	Y	Y	Y	Y	Y	Y	Y	Y	Y	Y	Y	Y	Y	Y	Y	Y
2. Was the selection of study subjects/patients free from bias?	N	Y	N	Y	N	N	N	Y	Y	Y	Y	Y	Y	Y	Y	Y	Y	Y	Y	Y	Y	N	N
3. Were study groups comparable?	Y	Y	Y	Y	Y	Y	Y	Y	Y	Y	Y	Y	Y	Y	Y	Y	Y	Y	Y	Y	Y	Y	Y
4. Was method of handling withdrawals described?	Y	N	Y	Y	Y	Y	Y	Y	Y	Y	Y	Y	Y	Y	Y	N	N	Y	Y	Y	Y	Y	Y
5. Was blinding used to prevent introduction of bias?	N	Y	Y	Y	Y	Y	Y	Y	Y	Y	Y	Y	Y	Y	Y	N	Y	Y	Y	Y	Y	Y	N
6. Were intervention /exposure factor or procedure and any comparison(s) described in detail?	Y	Y	Y	Y	Y	Y	Y	Y	Y	Y	Y	Y	Y	Y	Y	N	Y	Y	Y	Y	Y	Y	Y
7. Were outcomes clearly defined and the measurements valid and reliable?	Y	Y	Y	Y	Y	Y	Y	Y	Y	Y	Y	Y	Y	Y	Y	Y	Y	Y	Y	Y	Y	Y	Y
8. Was the statistical analysis appropriate for the study design and type of outcome indicators?	Y	Y	Y	Y	Y	Y	Y	N	Y	Y	Y	Y	Y	Y	Y	Y	Y	Y	Y	Y	Y	Y	Y
9. Were conclusions supported by results with biases and limitations taken into consideration?	Y	Y	Y	Y	Y	Y	Y	Y	Y	Y	Y	Y	Y	Y	Y	Y	Y	Y	Y	Y	Y	Y	Y
10. Is bias due to study's funding or sponsorship unlikely?	Y	Y	Y	Y	Y	Y	Y	Y	Y	Y	Y	Y	Y	Y	Y	Y	Y	Y	Y	Y	Y	Y	Y
**OVERALL QUALITY**	Nº	P	Nº	P	Nº	Nº	Nº	P	P	P	P	P	P	P	P	Nº	P	P	P	P	P	Nº	Nº

[1] American Dietetic Association Quality Criteria Checklist for Primary Research (22)
[2] Y, yes; N, no
[3] P, positive rating; Nº, neural rating

Supplementary Table S3. ***(continued)*** [1]

First author, year of publication (reference)	Santiago, 2013	Schmidt, 2004	Srinivasan, 2017	Tamura, 2003	Tofail, 2006	Tofail, 2008	Van goor, 2011	Vuori, 1979	Waber, 1981	Zhou, 2006	Zhou, 2015
**VALIDITY QUESTIONS**											
1. Was the research question clearly stated?	Y	Y	Y	Y	Y	Y	Y	Y	Y	Y	Y
2. Was the selection of study subjects/patients free from bias?	N	Y	Y	Y	N	Y	Y	N	N	Y	Y
3. Were study groups comparable?	Y	Y	N	Y	N	Y	Y	Y	Y	Y	Y
4. Was method of handling withdrawals described?	N	N	Y	N	Y	Y	Y	N	N	Y	Y
5. Was blinding used to prevent introduction of bias?	Y	Y	Y	Y	Y	Y	Y	N	N	Y	Y
6. Were intervention /exposure factor or procedure and any comparison(s) described in detail?	Y	Y	Y	Y	Y	Y	Y	Y	Y	Y	Y
7. Were outcomes clearly defined and the measurements valid and reliable?	Y	Y	Y	Y	Y	Y	Y	Y	Y	Y	Y
8. Was the statistical analysis appropriate for the study design and type of outcome indicators?	Y	Y	Y	Y	Y	Y	Y	Y	Y	Y	Y
9. Were conclusions supported by results with biases and limitations taken into consideration?	Y	Y	Y	Y	Y	Y	Y	Y	Y	Y	Y
10. Is bias due to study's funding or sponsorship unlikely?	Y	Y	Y	Y	Y	Y	Y	Y	Y	Y	Y
**OVERALL QUALITY**	Nº	P	Nº	P	Nº	P	P	Nº	Nº	P	P

[1] American Dietetic Association Quality Criteria Checklist for Primary Research (22)
[2] Y, yes; N, no
[3] P, positive rating; Nº, neural rating

Supplementary Table S4. Summary of nutritional intervention for the included studies

	OCED[1]	Type of nutritional intervention
Brei, 2017	1	Nutrient supplement + dietary counselling
Catena, 2016	1	Fortified food
Caulfield, 2010	2	Nutrient supplement
Cheatham, 2012	1	Nutrient supplement
Chang, 2013	3	Nutrient supplement
Christian, 2010	4	Nutrient supplement
Christian, 2016	4	Nutrient supplement
Dunstan, 2008	1	Nutrient supplement
Gould, 2014	1	Nutrient supplement
Hamandani, 2002	4	Nutrient supplement
Hanieh, 2013	3	Nutrient supplement
Helland, 2001	1	Nutrient supplement
Helland, 2003	1	Nutrient supplement
Helland, 2008	1	Nutrient supplement
Hurtado, 2015	1	Fortified food
Joos, 1983	2	Nutrient supplement
Judge, 2007	1	Fortified food
Li, 2009	2	Nutrient supplement
Makrides, 2010	1	Nutrient supplement
McGrath, 2006	4	Nutrient supplement
Mulder, 2014	1	Nutrient supplement
Prado, 2012	3	Nutrient supplement
Ramakrishnan, 2016	2	Nutrient supplement
Santiago, 2013	1	Fortified food
Schmidt, 2004	4	Nutrient supplement
Srinivasan, 2017	3	Nutrient supplement
Tamura, 2003	1	Nutrient supplement
Tofail, 2006	4	Nutrient supplement
Tofail, 2008	4	Food + nutrient supplements
Van goor, 2011	1	Nutrient supplement
Vuori, 1979	2	Food + nutrient supplements
Waber, 1981	2	Food + nutrient supplements + education
Zhou, 2006	1	Nutrient supplement
Zhou, 2015	1	Nutrient supplement

[1] OCED, the organisation for economic co-operation and development criteria; 1=high income country, 2=higher middle income country, 3=lower middle income country & 4=low income country

Supplementary Table S5. Cognitive assessment tests used by the included studies

**Type of assessment**	**Test name**	**Description**	**Age range**	**Time to administer**
Overall ability	Bayley Scales of Infant Development, First Edition (BSID-I) (Bayley, 1969)	Two administrated scales: mental, motor and behaviour. Raw scores can be converted to scale scores and then composite scores.	2 to 30 mths	45 to 60 mins.
Overall ability	Bayley Scales of Infant Development, Second Edition (BSID-II) (Bayley, 2005)	Five administrated scales: cognitive, language and motor, socio-emotional and adaptive behaviour. Composite scores, percentile ranks and confidence intervals can be calculated from the raw scores obtained for each scale.	1 to 42 mths.	30 to 90 mins.
Overall ability	Bayley Scales of Infant Development, Third Edition (BSID-III)	Three administrated scales: mental, motor and behaviour rating. The Mental Development Index (MDI) is derived from the raw score record from the mental scale. The Psychomotor Development Index (PDI) is derived from the raw scores recorded from the motor scale. The behaviour rating scale provides a qualitative assessment (using percentiles) of factors including orientation/engagement, emotional regulation and motor quality.	1 to 42 mths.	25 to 60 mins.
Overall ability	British Ability Scales, Second Edition (BAS-2) (Elliot, 1996)	Three administrated scales: verbal ability, non-verbal reasoning ability and spatial ability. Scores can be expressed as ability scores, standardised scores and composite ability score (General Conceptual Ability Score (GCA).	3 to 17.11 yrs.	30 to 45 mins.
Overall ability	Child Development Inventory (Brandstetter et al., 2002)	Measures development in eight areas: social, self-help, gross motor, fine motor, expressive language, language comprehension, letters, and numbers. It also includes a General Development Scale.	1.3 to 6 yrs.	30 to 50 mins.

Supplementary Table S5. *(Continued)*

**Type of assessment**	**Test name**	**Description**	**Age range**	**Time to administer**
Overall ability	Differential Ability Scales, First edition (DAS) (Elliott, 1983)	Seventeen cognitive and three achievement subtests. The Preschool-aged level is classified into three ability areas: verbal ability, nonverbal reasoning ability and diagnostic subtest. The School-aged is classified into four ability areas: verbal ability, nonverbal reasoning ability, spatial ability and diagnostic subtest. Scores can be expressed as ability scores, standardised scores and overall composite score (GCA).	2.6 to 17.11 yrs.	35 to 90 mins.
Overall ability	Fagan Test of Infant Intelligence, Second Edition (FTII) (Fagan & Detterman, 1992)	Measures visual novelty preference and recognition memory. For each novelty test, the infant is exposed to a stimulus, for example a picture of a woman's face, the researcher sitting behind a computer records the duration of the infant's fixation on the stimulus. The next test, involves pairing the familiar stimulus with a new stimulus to evaluate the infant's recognition memory. The researcher once again records the fixation time from the infant. Ten stimulus pictures are presented to infants aged 6.5 to 12 months. A novelty preference score is calculated for each test by dividing the time spent focusing on the picture by the total amount of time looking at both stimuli. For each age a mean novelty preference score is calculated from the series of stimulus pictures.	27, 29, 39 and 52 postnatal wks.	6 to 60 secs.
Overall ability	Griffiths Mental Development Scales (GMDS 0-2) (Griffiths, 1970)	Five administrated subscales: locomotor, personal-social, language, eye-and-hand coordination and performance. Raw subscale scores can be converted to standardised scores: age equivalents, sub-quotients and general quotients and percentile equivalents.	0 to 2 yrs.	50 to 60 mins.

Supplementary Table S5. *(Continued)*

Type of assessment	Test name	Description	Age range	Time to administer
Overall ability	Kaufman Assessment Battery for Children, Second Edition (KABC-II) (Kaufman & Kaufman, 1983)	Five administrated scales: simultaneous, sequential, planning, learning and knowledge. Provides Age-based standard scores, age equivalents, and percentile ranks. Two global scores can also be determined: Mental Processing Index (MPI) and Fluid-Crystallized Index (FCI).	3 to 18 yrs.	25 to 70 mins.
Overall ability	McCarthy Scales of children's Abilities (MSCA) (McCarthy, 1972)	Six administrated scales: verbal, perceptual-performance scale, quantitative, composite (general cognitive), memory and motor. Six scaled scores are attained and a composite score (General Cognitive Index) is also derived.	2.5 to 8.5 yrs.	45 to 60 mins.
Overall ability	Mullen Scales of Early Learning (MSEL) (Mullen, 1995)	Five administrated scales: fine and gross motor function, visual reception, expressive and receptive language. The scoring available includes percentiles, and age-equivalents for each scale and the Early Learning Composite score.	0 to 68 mths.	15 to 60 mins.
Overall ability	The Stanford-Binet Intelligence Scale, Fourth edition (SB4) (Thorndike, Hagen, & Sattler, 1986)	Four administrated scales: verbal reasoning, visual reasoning, quantitative reasoning and short-term memory. Raw scores can be converted into three types of standard scores: standard age scores, scale specific scores and test composite score.	2 to 85 yrs.	20 mins.
Overall ability	Wechsler Preschool and Primary Scale of Intelligence, Third Edition (WPPSI-III) (Wechsler, 2002)	Five administrated scales: Verbal IQ (VIQ), Performance IQ (PIQ), Full Scale IQ (FSIQ), Processing Speed (PSQ) and General Language Composite (GLC). Provides scaled scores by age, as well as verbal, performance and full scale IQ scores. The processing speed quotient can be derived for children aged 4 to 7.3 years and general language composite can be determined for 2.6 years to 7.3 years.	2.6 to 7.3 yrs.	30 to 60 mins.

Supplementary Table S5. *(Continued)*

Type of assessment	Test name	Description	Age range	Time to administer
Overall ability	Universal Non-verbal Intelligence Test, first edition (UNIT-1) (Bracken & McCallum, 1998)	Administrates six subtests assessing symbolic memory (0-30 possible raw score points), cube design (0-53), spatial memory (0-27), analogic reasoning (0- 31), object memory (0-30), and mazes (0-93).	5 to 17.11 yrs.	30 to 45 mins.
Multiple cognitive domains	A Developmental Neuropsychological Assessment, Second Edition (NEPSY-II) (Korkman, Kirk, & Kemp, 1998)	Measures six cognitive domains: executive function, language, memory and learning, sensorimotor, visual spatial processing and social perception. Scores can be expressed as standardised scores, percentile ranks, cumulative percentages, or a normative sample percentage.	3 to 16 yrs.	45 to 60 mins.
Multiple cognitive domains	Wide Range Achievement Test, Fourth Edition (WRAT4) (Jastak & Wilkinson, 1984)	Assess academic skills: reading, mathematics and nonverbal reasoning ability. Standard scores, percentile ranks, stanines, normal curve equivalents, grade equivalents, and Rasch ability scaled scores can be determined from test results.	5 to 94 yrs.	15 to 45 mins.
Executive Function	Go/no-go task (Konishi, Nakajima, Uchida, Sekihara, & Miyashita, 1998)	A computerized task in which a stimuli is presented and the participant is required to make a response by either pressing the ‘go’ computer button or not pressing the go button (no-go). The participant’s task accuracy and reaction times are measured for each stimuli to provide an overall assessment of inhibitory control.	Not specified.	Not specified.
Executive Function	Snack Delay Test (Carlson, 2005; Kochanska, Murray, & Harlan, 2000)	The child, waits for the researcher to ring the bell before retrieving a snack under a transparent cup. The following trials of delayed time are used: 10 to 30 seconds. If the child retrieved and ate the snack before the allocated time, the duration of the waiting time before eating the snack was	Not specified.	Not specified.

		recorded. The average amount of waiting time before eating the snack is calculated across the 4 trials.		

Supplementary Table S5. *(Continued)*

**Type of assessment**	**Test name**	**Description**	**Age range**	**Time to administer**
Executive Function	The Stroop test (Bull & Scerif, 2001)	The inhibitory control test requires participants to name the colour of the printed letters which are written in a colour that does not correspond with the word.	Not specified.	Not specified.
Executive function	Window Test (Russell, Mauthner, Sharpe, & Tidswell, 1991)	The assessor places a treat inside 1 of 2 boxes, both these boxes have a clear window which allows the child to see the treat. The child is asked to identify the box which has the treat inside and then obtain the treat. After, two trials the rules are reversed, the child is asked to identify the box without the treat and then instructed to obtain the box. The score is calculated based on the number of correct trials after the rule was reversed, out of 6 trials.	Not specified.	Not specified.
Language	Bear activity story (Fein, 1995; Morrow, 1986)	The purpose of the activity is to assess children's language development and narrative ability. A child is given props in a particular order and allowed to play for 10 mins. The child is then asked to tell a story with these given props. Based on the cohesion and complexity of the story, a score is derived from this activity.	Not specified.	Not specified.
Language	British Picture Vocabulary Scale, Second Edition (BPVS-II) (L. M. Dunn, Dunn, Whetton, & Burley, 1997)	The scale assesses receptive vocabulary. A spoken word is said to a child, after hearing the word they must choose the picture that best illustrates the meaning of the word from four options. The words provided represent a broad range of topics including actions, animals, toys, emotions etc. A score is derived from the number of correct answers. The total raw score can be converted to a percentile rank, mental age, or a standard deviation IQ score.	3 to 15.11 yrs.	10 mins.

Supplementary Table S5. *(Continued)*

Type of assessment	Test name	Description	Age range	Time to administer
Language	MacArthur-Bates Communicative Development Inventories (CDI) (Fenson et al., 2007)	Provides parent/caregiver report forms for assessing language and communication. There are three available inventories: 1) words and gestures (used for children 8–18 mths) 2) words and sentences (used for children 16–30 mths) 3) expressive vocabulary and grammar (for use with children 30–37 mths). The inventory yields percentile scores by month for most subscales, as well as a composite score.	8 to 37 mths.	30 to 45 mins.
Language	Peabody Picture Vocabulary Test, Third Edition (PPVT-III) (L. M. Dunn & Dunn, 1981)	Test is used to measure receptive vocabulary. A spoken word is said to a child, after hearing the word they must choose the picture that best illustrates the meaning of the word from four options. A score is derived from the number of correct answers. Raw scores can be converted to age-based standard scores, percentiles, normal curve equivalents (NCEs), stanines, and age equivalents.	2.6 to 90 yrs.	10 to 15 mins.
Language	Word-object pairing associate learning task (Pegg & Werker, 1997; Stager & Werker, 1997; Werker & Lalonde, 1988)	The task assesses the infant's ability to make an association between a word and object. The task is performed in two trial rounds: 1) the infant is present with word A and object A in a sequential order; 2) the infant is presented with word A and then object B in an unrelated order. A longer looking time by the infant in trial 2 suggests that the relationship between the word and object has been established. The mean looking time is established for each infant.	8 to 14 mths.	Not specified.
Attention	Attention Network Test (Fan, McCandliss, Sommer, Raz, & Posner, 2002)	The test assesses three attention networks (executive, alerting, and orienting) using a single behavioural task. The test requires a child to feed a fish (presented on a computer screen) by pressing a button that corresponds to the direction of the central fish. The child's efficiency score is calculated	6 to 10 yrs.	25 to 30 mins.

		for reaction times and error percentage for three blocks of 48 trials.		

Supplementary Table S5. *(Continued)*

**Type of assessment**	**Test name**	**Description**	**Age range**	**Time to administer**
Attention	Conners'Kiddie Continuous Performance Test, second edition (K-CPT) (Conners, 2000)	The test is designed to assess attention deficits using pictures of objects they are familiar to young children. The participant is required to respond to targets and withhold from responding to non-targets presented on the computer screen. Overall performance is measured based on four different aspects of attention including inattentiveness, impulsivity, sustained attention and vigilance.	4 to 7 yrs.	7.5 mins.
Attention	Single object task (Colombo et al., 2004; Kannass, Colombo, & Carlson, 2009)	The task measures the child's ability to sustain their attention on a toy without any distraction or competition. The task is recorded on a video camera for scoring. The coders calculates: the total duration of looking, total number of episodes of inattention, average length of looks to the toys, and total number of looks to the toys.	Not specified.	Not specified.
Attention	Distractibility task (Colombo et al., 2004; Kannass et al., 2009)	The task measure's the child's ability to maintain their attention on a particular object in the presence of a distractions. The task is recorded on a video camera for scoring. The coders calculates: the total duration of looking, total number of episodes of inattention, average length of looks to the toys, and total number of looks to the toys.	Not specified.	Not specified.
Attention	Multiple object task (Colombo et al., 2004; Kannass et al., 2009)	The task measure the child's ability to sustain attention one toy with the distraction of others toys competing for their attention. The task is recorded on a video camera for scoring. The coders calculates: the total duration of looking, total number of episodes of inattention, average length of looks to the toys, and total number of looks to the toys.	Not specified.	Not specified.

Supplementary Table S5. *(Continued)*

Type of assessment	Test name	Description	Age range	Time to administer
Memory	A-not-B task (Sparrow, Balla, & Cicchetti, 1984)	The task is designed to assess an infant's working memory. The research hides a particular item in location A and distracts the child for 3 seconds before they are able to retrieve the item. The task is repeated, except the item is hidden in location B and the child is distracted for 10 seconds before they can retrieve the item. The child's performance accuracy is measured by viewing a video recording frame by frame. A shorter distance between the hidden toy and the location where the child searched illustrates better performance accuracy and therefore working memory.	7 to 12 mths.	Not specified.
Problem solving	2- Step problem solving task (Willatts, 1984a, 1984b, 1989, 1997, 1999)	Tests an infant's ability to execute a series of steps to retrieve a toy. The test scores two separate steps: the infant's ability to pull a toy within reach and find a hidden toy. The 2-step problem solving task is presented five times to the infant. In each trial, 3 behaviours were evaluated: cloth behaviour (the way the child handled the cloth), fixation behaviour (the way the child fixed his or her vision on the toy) and toy behaviour (the way the child grasped the toy). The behaviours were scored on a 3 point scale: 0 for no evidence of intention, 1 for possible intention and 2 for clear evidence of intention. The scores for each behaviour were added to give the total intention score for each trial. The score from each trial from was summed together to provide the total score.	6 to 8 mths.	Not specified.
Concept formation	Goodenough & Harris Draw-A Person Test,	To evaluate the child's concept formation ability based on their drawing of the human figure. The drawing is scored	Not specified.	Not specified.

	revised version (Harris, 1963)	according to the presence or absence of specific features, for example head, hair, eyes etc. Scores ranged from 0 to 20.		

Supplementary Table S5. *(Continued)*

**Type of assessment**	**Test name**	**Description**	**Age range**	**Time to administer**
Auditory processing	Illinois Test of Pycholinguistic Abilities: Auditory Sequential Memory (ASM) (Kirk, McCarthy, & Kirk, 1968)	Measures the ability to orally reproduce a series of digits. The number of digits presented increases for each test. The raw score obtained from the test can be converted to age-equivalent scores.	2 to 10 yrs.	Not specified.
Visual processing	Illinois Test of Pycholinguistic Abilities: Visual Sequential Memory (VSM) (Kirk et al., 1968)	A chip design sequence is presented to a child for five seconds and then removed. The child is then asked to recreate the observed sequence of chips from memory. The sequence increases in length from two chip design up to an eight chip design. The raw score obtained from the test can be converted to age-equivalent scores.	2 to 10 yrs.	Not specified.
Visual processing	Imitation paradigm (Meltzoff & Moore, 1983)	To measure the long-term memory of infants, an action is demonstrated by a research which is then imitated by the infant after a delayed period of time. A designated number of different actions are demonstrated to the child. The responses imitation task is recorded via a video camera. The task is scored based on the number of successful intimated actions completed by the child.	18 to 24 mths.	Not specified.
Visual processing	The Knox Cube test (Stone & Wright, 1980)	The Knox Cube test is an index of attention span and short term memory. The child is directed to tap 4 cubes in a specific order, starting with the easiest sequence ‘1-4’ to the most challenging sequence ‘4-1-3-4-2-1-4’. Each dice is given a score of one for the correct tap and zero for the incorrect tap. A total score is calculated from the task.	3 yrs. and above	2 to 5 mins.

Supplementary Table S5. *(Continued)*

Type of assessment	Test name	Description	Age range	Time to administer
Visual processing	Visual attention (Birns, Barten, & Bridger, 1969; Friedman, 1972; Greenberg, O'Donnell, & Crawford, 1973)	Infants are presented with a series of visual stimulus card which display a variety of different patterns. The infant's duration and number of fixations on the stimulus is recorded using timer by an observer. A score is calculated from the completed trials using the stimulus cards. The number of frets/cries and movements is also recorded from the researcher that presents the stimulus. Habituation is defined as a decrement in fixation time between trial 1 (T1) and trial 8 (T8). The habituation rate is measured as the fraction of fixation time at T1 persisting at T8 (T1-T8/T1). Negative rates are counted as zero and scores range from 0 to 1.0.	Not specified.	Not specified.
Visual processing	Visuospatial Memory Delayed Response Task (Brody, 1981; Diamond & Doar, 1989; Pelphrey et al., 2004; Schwartz & Reznick, 1999)	For this task, an infant watches a researcher hide an object in a well with a covered lid. After a period of delay the infant is directed to find the object. The task is repeated a number of times until the infant loses interest. Calculation are made based on the number of times the cover is opened from the well.	Not specified.	Not specified.
Motor skills	Escalona and Corman's Albert Einstein Scales of Sensorimotor Development (Corman & Escalona, 1969)	Measures three subscales of sensorimotor functioning: prehension, object permanence and spatial relationships. The subscale prehension has 16 items which scores as stage 2 or 3 based on the infant's ability. The object permanence has 18 items which are scored as stage 3-6. The spatial relationships has 21 items and is scored as stages 3-6.	9 to 27 mths.	Not specified.
Motor skills	Finger-tapping test (Reitan & Wolfson, 1985)	The test measures fine motor speed. Participants are required to tap a lever with their index finger. The reported score is the average number of taps per hand.	9 yrs. and above	10 to 15 mins.

Supplementary Table S5. *(Continued)*

Type of assessment	Test name	Description	Age range	Time to administer
Motor skills	Grooved Pegboard test (Wilson, Lacoviello, Wilson, & Risucci, 1982)	Manipulative dexterity test that requires complex visual-motor coordination. The aim of the test is position 25 pegs into uniquely shaped slots. The pegs must be positioned correctly before they can be inserted into the hole. Scoring is based on the following factors: 1) the time taken to complete the test; 2) number of times the pegs are dropped; 3) the number of pegs inserted correctly. The total score is the sum of these three factors.	5 yrs. and above	10 to 15 mins.
Motor skills	Lower mirror movements (MM) (Hermsdorfer, Mai, & Marquardt, 1992)	The neurological development test measures 'involuntary movements of one body part that mirror the voluntary movement of the contralateral homologous body part'(Cincotta & Ziemann, 2008). The test compares the hand grip strength of the active hand versus the mirrored hand.	Not specified.	Not specified.
Motor skills	Movement Assessment Battery for Children (MABC) (Henderson & Sugden, 1992)	Measures manual dexterity, ball skills and static and dynamic balance in specific age categories.	4 to 12 yrs.	20 to 35 mins.
Motor skills	Peabody Developmental Motor Scales and Activity Cards (PDMS) (Folio & Fewell, 1983)	Consists of two subscales; gross and fine motor. The gross motor subscale, measure skills including reflex, balance, non-locomotion, locomotion and receipt and propulsion of objects. The fine motor subscale assesses grasp, eye-hand coordination, and hand use and hand dexterity. The following scores can be derived from the scale: age equivalents, percentile ranks, standard score or developmental motor quotient.	0 to 83 mths.	60 mins.

Supplementary Table S5. *(Continued)*

Type of assessment	Test name	Description	Age range	Time to administer
Behaviour	Ages and Stages Questionnaire, 2nd edition (ASQ) (Squires, Potter, & Bricker, 1999)	Parent or care giver completed screening questionnaire divided into the following domains: communication, gross motor, fine motor, problem-solving and personal adaptive skills. A pass or fail score is derived for all domains.	4 to 60 mths.	10 to 15 mins.
Behaviour	Behavioural Assessment System for Children, edition 2 (BASC-2) (Elliot, 1996)	Instruments designed to help diagnose and treat behavioural problems in children, adolescents and young adults. There are multiple forms to different age groups which allow parents, teachers and examinees to rate their behaviour using a specific point system.	2 to 21.11 yrs.	10 to 30 mins.
Behaviour	Brief Infant Toddler Social and Emotional Assessment (BITSEA) (Briggs-Gowan & Carter, 2002)	Screening test for identifying behavioural and emotional problems in infants and children. There two assessment forms: 1) is designed to be completed by parents or caregivers 2) is designed for professionals to complete. All questions are rated using a 3 point scale (0=not true/rarely, 1=somewhat true/sometimes, 2=very true/always). For certain items a respondent may also respond “N” which means “no opportunity.” A competence score a calculated based on the sum of the item ratings and a problem score is derived from the sum of the problem item ratings.	12 to 36 mths.	7 to 10 mins.
Behaviour	Child Behaviour Checklist (CBCL) (Achenbach, 1991)	A checklist designed to detect emotional and behavioural problems in children. The checklist consists of 100 questions, which is completed by parents of care givers. Responses are recorded on a Likert scale: 0= Not True, 1=somewhat true or sometimes true, 2= very true or often true. The scoring provides a summary and syndrome profile and five different Diagnostic and Statistical Manual-oriented scales.	1.5 to 5 yrs.	15 to 20 mins.

Supplementary Table S5. *(Continued)*

Type of assessment	Test name	Description	Age range	Time to administer
Behaviour	Friendship interview (Selman, 1980 ; Serafica, 1982)	This interview is used to assess a child's interpersonal understanding. The child is asked open and close-ended questions about their best friend and general friendship in the context of a pretend play date. Question answers are given the following codes: no friendship concept (0), physical interaction friendship (1), one-way friendship (2), fair-weather friendship (3), mutual relation (4), and autonomous interdependence friendship (5). An overall score is calculated from this coding system.	Not specified.	Not specified.
Behaviour	Preschool Behaviour Questionnaire (PBQ) (Behar & Stringfield, 1974)	The questionnaire is a 30-item instrument which assesses the child's behaviour problems. The items are divided into three scales: hostile-aggressive (11 items), anxious-fearful (9 items) and hyperactive-distractible (4 items). The mother rates items on a 3-point scale (0-2) to identify the frequency of behaviour problems. A syndrome score is derived from the sum of the related items. The syndrome score can also be used to calculate the internalizing and externalizing problem scale scores. The total score from all items is also calculated. Tables are also provided to determine if the syndrome score, problem scale score and total score represent normal, border line or clinical behaviour.	3 to 6 yrs.	5 to 10 mins.

Supplementary Table S5. *(Continued)*

Type of assessment	Test name	Description	Age range	Time to administer
Behaviour	Socioemotional Development Scale (Prado et al., 2010)	Scale was developed based on the Brief Infant-Toddler Social and Emotional Assessment. The scale is in an interview format, the child's parent or child giver rates 29 items probing specific aspects of child's behaviour on a scale from 0 to 2 (0= not true/rarely, 1=somewhat true/sometimes, 2= very true/often). Both competence items and problem items are given. A competence score a calculated based on the sum of the item ratings and a problem score is derived from the sum of the problem item ratings.	22 to 55 mths.	Not specified.
Behaviour	Strength and Difficulties Questionnaire (SDQ) (Goodman, 1999)	Brief screening questionnaire to assess behaviour in children. The questionnaire is divided into 5 scales: emotional symptoms, conduct problems, hyperactivity/inattention, peer relationship problems and prosocial behaviour. Each scale is scored from 0-10 and the total from four scales (emotional, conduct, hyperactivity and peer problems) can generate a total difficulty score (range 0-40). The total score from the emotional and peer items produces the internalising problems score (range 0-20) and the externalising score is extracted from the total score attained in the conduct and hyperactivity items.	3 to 16 yrs.	10 mins.
Behaviour	Vineland's Adaptive Behaviour Scales, second edition (Vineland II) (Sparrow et al., 1984)	Measures the child's current level of adjustment and functioning. The parents are required to indicate the child's level of skill in 4 major domains: communication, daily living skills, socialization and motor behaviour. There is an additional form available for school teachers to evaluate a child's behaviour. The scale provides the following scores:	0 to 90 yrs.	20 to 60 mins.

		Domains and Adaptive Behavior Composite, percentile ranks, Adaptive levels and age equivalents.		

Supplementary Table S5. *(Continued)*

**Type of assessment**	**Test name**	**Description**	**Age range**	**Time to administer**
Behaviour	Wolke's Behaviour Scale (modified version) (Wolfe, Skuse, & Mathisen, 1990)	Five scaled tool designed to be completed by the mother which measures infant behaviour. All five scales have a 9 point rating system: infant's activity (very still=1 to over-active=9), emotional tone (unhappy=1 to extremely happy=9), responsiveness to examiner in the first 10 minutes (avoiding=1 to inviting=9), cooperation with the test procedure (resists all suggestions=1 to always complies=9), and vocalization (very quiet=1 to constantly vocal=9). Based on the given ratings, a scale scores can be derived.	Not specified.	Not specified.

References

Achenbach, T. (1991). *Manual for the Child Behaviour Checklist 1.5- 5 and 1991 profile*. Burlington, VT: University of Vermont Department of Psychiatry.

Bayley, N. (1969). *Manual for Bayley Scales of Infant Development* (1st ed.). New York, NY Psychological Corp.

Bayley, N. (2005). *Bayley Scales of Infant Development* (3rd ed.). San Antorio, TX: Harcourt Assessment.

Behar, L., & Stringfield, S. (1974). A behaviour rating scale for the preschool child *Developmental Psychology, 10*, 601-610.

Birns, B., Barten, S., & Bridger, W. (1969). Individual differences in temperamental characteristics of infants *Trans, 31*.

Bracken, B. A., & McCallum, R. S. (1998). *Universal Non-verbal Intelligence Test (UNIT)*. Austin, TX: PRO-ED.

Brandstetter, G., Siebler, V., Schneider, H., Grässle, A., Steinmacher, J., & Bode, H. (2002). Elternfragebogenzur Entwicklung im Kleinkindalter (EFkE) - ein Screeninginstrument: I. Normierung. *Kinderarztl Prax, 5*, 338-344.

Briggs-Gowan, M., & Carter, A. (2002). *Brief Infant-Toddler Social and Emotional Assessment (BITSEA) Mannual, Version 2*. New Haven, CT: Yale University

Brody, L. (1981). Visual short-term cued recall memory in infancy. *Child Development, 52*, 242-250.

Bull, R., & Scerif, G. (2001). Executive functioning as a predictor of children's mathematics ability: Inhibition, switching, and working memory. *Developmental Neuropsychology, 19*(3), 273-293.

Carlson, S. M. (2005). Developmentally sensitive measures of executive function in preschool children *Developmental Neuropsychology, 28*(2), 595-616.

Cincotta, M., & Ziemann, U. (2008). Neurophysiology of unimanual motor control and mirror movements. *Clin Neurophysiol, 119*(4), 744-762.

Colombo, J., Kannass, K., Shaddy, D., Kundurthi, S., Maikranz, J., Anderson, C. J., et al. (2004). Maternal DHA and the development of attention in infancy and toddlerhood. *Child Development, 75*, 1254-1267.

Conners, C. K. (2000). *Conners' Continuous Performance Test II: Computer program for Windows technical guide and software manual*. Tonawanda, NY: Multi-Health Systems Inc.

Corman, H., & Escalona, S. (1969). Stages of sensorimotor development: a replication study *Merrill-Palmer Quarterly, 15*, 351-361.

Diamond, A., & Doar, B. (1989). The performance of human infants on a measure of frontal cortex function, delayed response task. *Developmental Psychology, 22*, 271-294.

Dunn, L. M., & Dunn, L. (1981). *Peabody Picture Vocabulary Test- revised (PPVT-R)*. Pine Circle, MN: American Guidance Service

Dunn, L. M., Dunn, L., Whetton, C., & Burley, J. (1997). *British Picture Vocabulary Scale* (2nd ed.). London, UK: NFER-Nelson Publishing Co, Ltd.

Elliot, C. (1996). *British Ability Scales* (2nd ed.). London, UK: NFER-Nelson Publishing Co, Ltd.

Elliott, C. D. (1983). *Differential Ability Scales Mannual 2: Technical Handbook* Windsor Berkshire, England NFER-Nelson

Fagan, J., & Detterman, D. (1992). The Fagan test of infant intelligence: a technical summary. *The Journal of Applied Developmental Psychology, 13*, 173-193.

Fan, J., McCandliss, B. D., Sommer, T., Raz, A., & Posner, M. I. (2002). Testing the efficiency and independence of attentional networks. *J Cogn Neurosci, 14*(3), 340-347.

Fein, G. G. (1995). Toys and stories In A. D. Pellegrini (Ed.), *The Future of Play Theory: A Multidisciplinary Inquiry into the Contributions of Brian Sutton-Smith* Albany, NY: State University of New York Press

Fenson, L., Marchman, V., Thal, D., Dale, P., Reznick, J., & Bates, E. (2007). *The MacArthur-Bates Communicative Development Inventories: User's Guide and Technical Manual* (2nd ed.). Baltimore, MD: Paul H. Brookes Publishing Co.

Folio, M., & Fewell, R. (1983). *Peabody Development Motor Scales and Acuity Cards* Allen, TX: DLM Teaching Resources.

Friedman, S. (1972). Newborn visual attention to repeated exposures to redundant vs. novel targets. *Perception & Psychophysics, 12*.

Goodman, R. (1999). Strengths and difficulties questionnaire.   Retrieved January 25th 2015, from http://www.sdqinfo.com/

Greenberg, D., O'Donnell, W., & Crawford, D. (1973). Complexity levels, habituation and individual differences in early infancy. *Child Development, 44*.

Griffiths, R. (1970). *The abilities of young children. A comprehensive system of mental measurement for the first eight years of life* London, UK: Child Development Research Centre.

Harris, D. (1963). *Children's Drawings as Measures of Intellectual Maturity: A Revision and Extension of the Goodenough Draw-a-Man Test* New York, NY: Harcourt, Brace and World.

Henderson, S. E., & Sugden, D. A. (1992). *Movement Assessment Battery for Children (MABC)* London, England: Psychological Corp.

Hermsdorfer, J., Mai, N., & Marquardt, C. (1992). Evaluation of precision grip using pneumatically controlled loads. *J Neurosci Methods, 45*(1-2), 117-126.

Jastak, S., & Wilkinson, G. (1984). *Wide Range Achievement Test (Revised)*. Wilmington, DE: Jastek Associates

Kannass, K., Colombo, J., & Carlson, S. (2009). Maternal DHA levels and toddler free-play attention *Developmental Neuropsychology, 34*, 159-174.

Kaufman, A., & Kaufman, N. (1983). *Kaufman Assessment Battery for Children (K-ABC)*. Circle Pines, MN: American Guidance Services

Kirk, S., McCarthy, J., & Kirk, W. (1968). *Illinois Test of Pycholinguistic Abilities (revised)* Champaign, IL: University of Illinois Press.

Kochanska, G., Murray, K., & Harlan, E. (2000). Eftortful control in early childhood: continuity and change, antecedents and implications for social development. *Developmental Psychology, 36*(2).

Konishi, S., Nakajima, K., Uchida, I., Sekihara, K., & Miyashita, Y. (1998). No-go dominant brain activity in human inferior prefrontal cortex revealed by functional magnetic resonance imaging. *European Journal of Neuroscience, 10*(3), 1209-1213.

Korkman, M., Kirk, U., & Kemp, S. (1998). *NEPSY: A Developmental Neuropsychological Assessment*. Orlando, FL: The Psychological Corporation.

McCarthy, D. (1972). *Manual for the McCarthy scales of children's abilities. The Psychological Corporation*. New York, NY: Harcourt Brace Jovanovich Inc.

Meltzoff, A., & Moore, M. (1983). Newborn infants imitate adult facial gestures. *Child Development, 54*, 702-709.

Morrow, L. (1986). Effects of structural guidance in story telling on children's dictation of original stories. *Journal of Reading Behavior, 18*.

Mullen, E. (1995). *Mullen Scales of Early Learning, AGS edition: manual and item assessment book*. Circle Pines, MN: American Guidance Service Inc.

Pegg, J., & Werker, J. (1997). Adult and infant perception of two English phones *The Journal of the Acoustical Society of America, 102*(37), 42-53.

Pelphrey, K., Reznick, J., Goldman, B. D., Sasson, N., Morrow, J., Donahoe, A., et al. (2004). Development of visuospatial short-term memory in the second half of the first year. *Developmental Psychology, 40*, 836-351.

Prado, E., Hartini, S., Rahmawati, A., Ismayani, E., Hidayati, A., Hikmah, N., et al. (2010). Test selection, adaptation, and evaluation: A systematic approach to assess nutritional influences on child development in developing countries. *British Journal of Educational Psychology, 80* 31-53.

Reitan, R. M., & Wolfson, D. (1985). *The Halstead-Reitan Neuropsychological Test Battery: theory & clinical interpretation* Tucson, AZ: Neuropsychology Press.

Russell, J., Mauthner, N., Sharpe, S., & Tidswell, T. (1991). The 'windows task' as a measure of strategic deception in preschoolers and autistic subjects. *The British Journal of Developmental Psychology, 9*(2), 331-349.

Schwartz, B., & Reznick, J. (1999). Measuring infant spatial working memory using a modified delayed-response procedure. *Memory, 7,* 1-17.

Selman, R. (1980). *The growth of interpersonal understanding: developmental and clinical analyses* New York, NY: Academic Press.

Serafica, F. C. (1982). Conceptions of friendship and interaction between friends: An Organismic-Developmental Perspective In F. C. Serafica (Ed.), *Social cognitive development in context* New York, NY: The Guilford Press.

Sparrow, S., Balla, D., & Cicchetti, D. (1984). *Vineland Adaptive Behavior Scales Survey Form Manual (Interview Edition)*. Circle Pines, MN: American Guidance Service.

Squires, J., Potter, L., & Bricker, D. (1999). *The Ages and Stages Questionnaire user's guide* (2nd ed.). Baltimore, MD Paul H. Brookes Publishing, Co.

Stager, C., & Werker, J. (1997). Infants listen more for phonetic detail in speech perception than in word-learning tasks. *Nature, 388,* 381-382.

Stone, M., & Wright, B. (1980). *Knox's Cube Test* Chicago, IL: Stoelting Company.

Thorndike, R., Hagen, E., & Sattler, J. (1986). *Stanford-Binet Intelligence Scale: Technical Manual* (4th ed.). Itasca, IL: The Riverside Publishing Company.

Wechsler, D. (2002). *Wechsler Preschool and Primary Scale of Intelligence* (3rd ed.). San Antonio, TX: Harvcourt Assessment Inc.

Werker, J., & Lalonde, C. (1988). Cross-language speech perception: initial capabilities and developmental change. *Developmental Psychology, 24,* 1-12.

Willatts, P. (1984a). The stage-IV infant's solution of problems requiring the use of supports *Infant Behavior & Development, 7,* 125-134.

Willatts, P. (1984b). Stages in the development of intentional search by young infants. *Developmental Psychology, 20,* 389-396.

Willatts, P. (1989). Development of problem-solving in infancy. In A. Slater & G. Bremner (Eds.), *Infant Development* London, UK Lawrence Erlbaum Associates Ltd

Willatts, P. (1997). Beyond the couch potato infant: how infants use their knowledge to regulate action, solve problems and achieve goals. In J. G. Bremmer, A. Slater & G. Butterworth (Eds.), *Infant Development: recent advances* (pp. 109-135). Hove, UK Psychology Press.

Willatts, P. (1999). Development of means-end behavior in young infants: pulling a support to retrieve a distant object *Developmental Psychology, 35,* 651-667.

Wilson, B., Lacoviello, J., Wilson, J., & Risucci, D. (1982). Purde Pegboard performance of normal children *Journal of Clinical Neurophysiology, 4,* 19-26.

Wolfe, D., Skuse, D., & Mathisen, V. (1990). Behavioral style in failure to thrive infants: a preliminary communication *Journal of Pediatric Psychology, 15,* 237-254.

Supplementary Figure S1. Review protocol
Effects of dietary interventions during pregnancy on infant and child cognitive outcomes: a systematic review and meta-analysis

Research question

Do nutrition interventions during pregnancy (exclusively) or pregnancy and lactation affect cognitive development in infants and young children
P [Population] - pregnant women (age age), offspring (aged 0-10 years)
I [Intervention] - dietary intervention (including; counselling, education, modify nutrient or calorie intake, nutrient supplementation, fortified foods)
C [Comparator] - Control group (placebo or alternate dietary intervention)
O [Outcome] – Cognitive outcomes of the offspring

Hypothesis (null)

Nutrition interventions during pregnancy (exclusively) or pregnancy and lactation does not change the cognitive development of infants and young children.

Outcome variable(s)

Global cognition
Quantitative Reasoning
Reading & Writing Ability
Short-Term Memory
Long-Term Storage and Retrieval
Attention
Visual processing
Auditory processing
Processing speed
Decision speed
Psychomotor skills
Behaviour

Predictors (explanatory variables)

Maternal diet, changing single macro –or micronutrient intake (e.g. fat intake – g/day or % of total energy intake) by:-
Modifying the whole diet
Modifying food group serves (i.e. fruit, vegetable)
Prescribing individual food/s (i.e. fish/seafood)
Prescribe fortified food product/s
Prescribe nutrient supplement/s with or without dietary modification

Confounding factors

Maternal socioeconomic indices, including income, education level
Maternal intelligence quotient (IQ)
Home stimulation
Ethnicity/Nationality
Smoking status
Body Mass Index (BMI) or weight (kg)
Income level of country (Organisation for Economic Co-operation and Development (OECD))

Databases to search

Embase
MEDLINE
Pre-MEDLINE
Proquest
Web of Science
CINAHL
Scopus
Cochrane
Maternity and Infant Care

Supplementary Figure S1. *(Continued)*

Keywords

Pregnancy
Cognition
Neurodevelopment
Infant
Child
Randomised control trial
Clinical trial
Food
Nutrition
Supplement

Inclusion criteria

No date limits
Human studies
Randomised or pseudorandomised controlled trials of any date
Pregnant women of any age or ethnicity
Singleton pregnancies
Dietary intervention/s, including dietary counselling and education as well as prescribed food/s, fortified foods or dietary supplements provided to pregnant women by any health professional
Measures cognitive outcomes of infants and children (less than 10 years) using cognitive assessment tests
Good or neutral methodological quality according to the Quality Criteria Checklist in the American Dietetic Association Evidence Analysis manual

Exclusion criteria

All other study designs including animal studies
Women that are not pregnant
Multiple births for the primary population
Dietary intervention/s are not provided to pregnant women
Dietary intervention/s that commence after pregnancy
Cognitive outcomes are not measured in children after pregnancy
Negative methodological quality according to the Quality Criteria Checklist in the American Dietetic Association Evidence Analysis manual

Data extracted

Study design
Study Aim
Number of participants [including; pregnant women and offspring]
Participant characteristics [including; offspring gender]
Maternal age, country, any demographic factors etc.
Intervention [including; duration i.e. pregnancy exclusively or pregnancy and lactation, marker of compliance i.e. blood test, check list]
Dietary nutrient of interest
Method of assessing cognition
Cognitive outcomes
Conclusion
Limitations

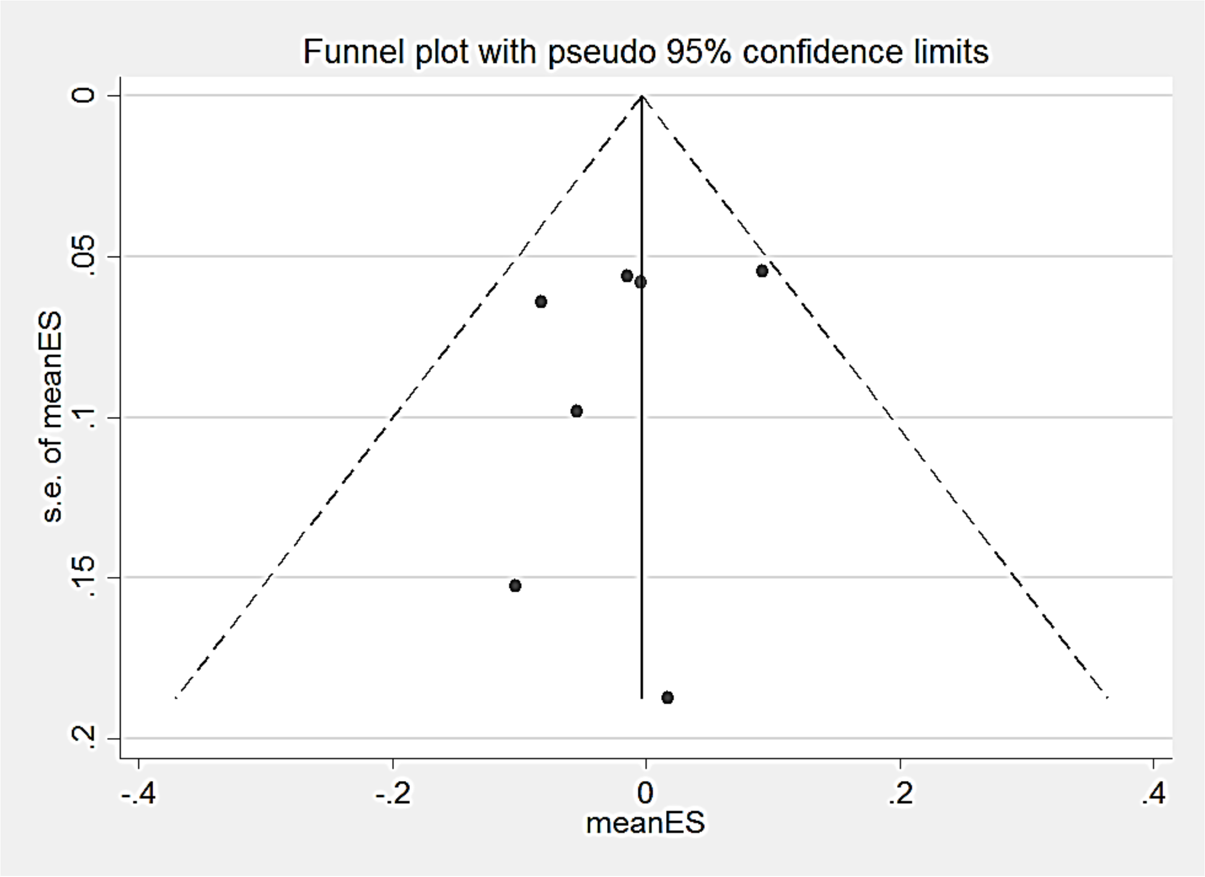

Supplementary Figure S2. Funnel plot for child behaviour outcomes with 95% confidence limits. meanSE, mean standard error.

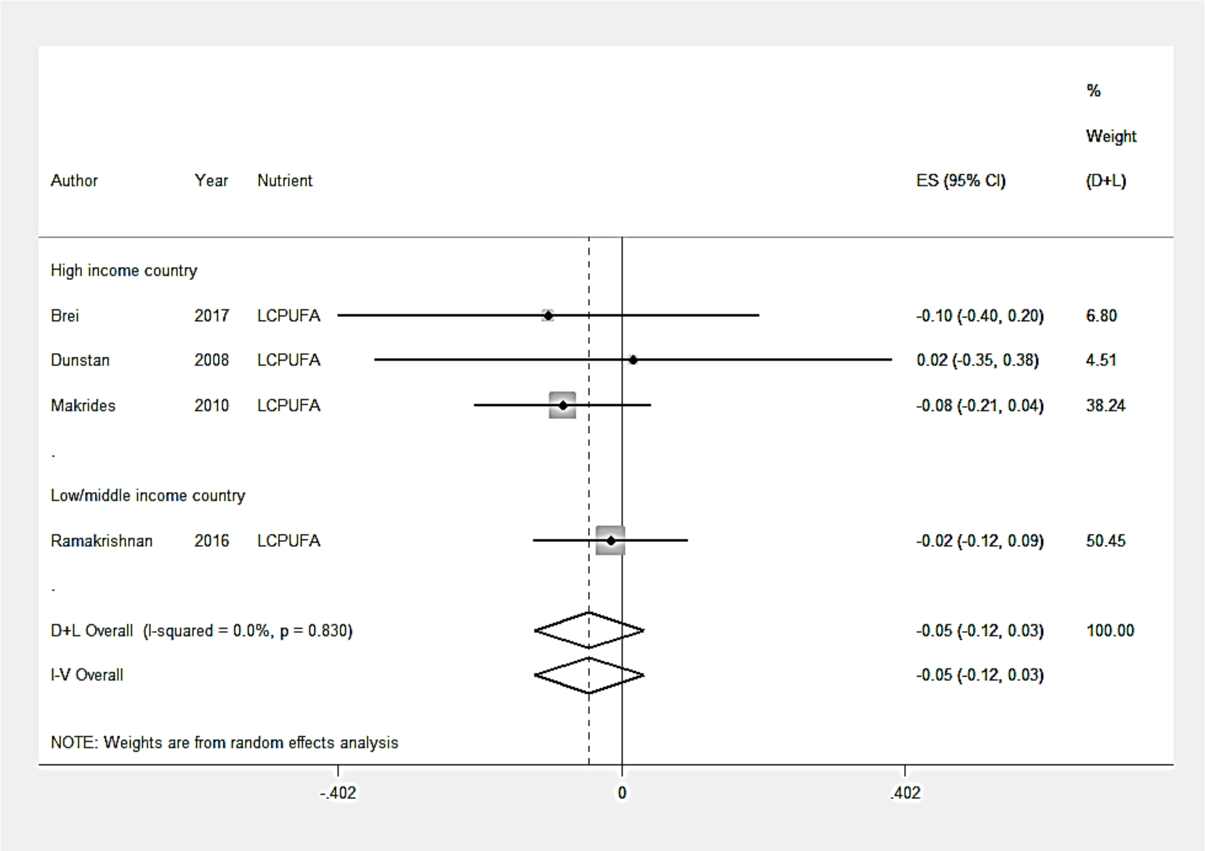

Supplementary Figure S3. Sensitivity analysis forest plot for child behaviour outcomes by country income. The overall effect size was estimated by standardised mean difference (SMD). Significance tests, high-income countries (P=0.25).

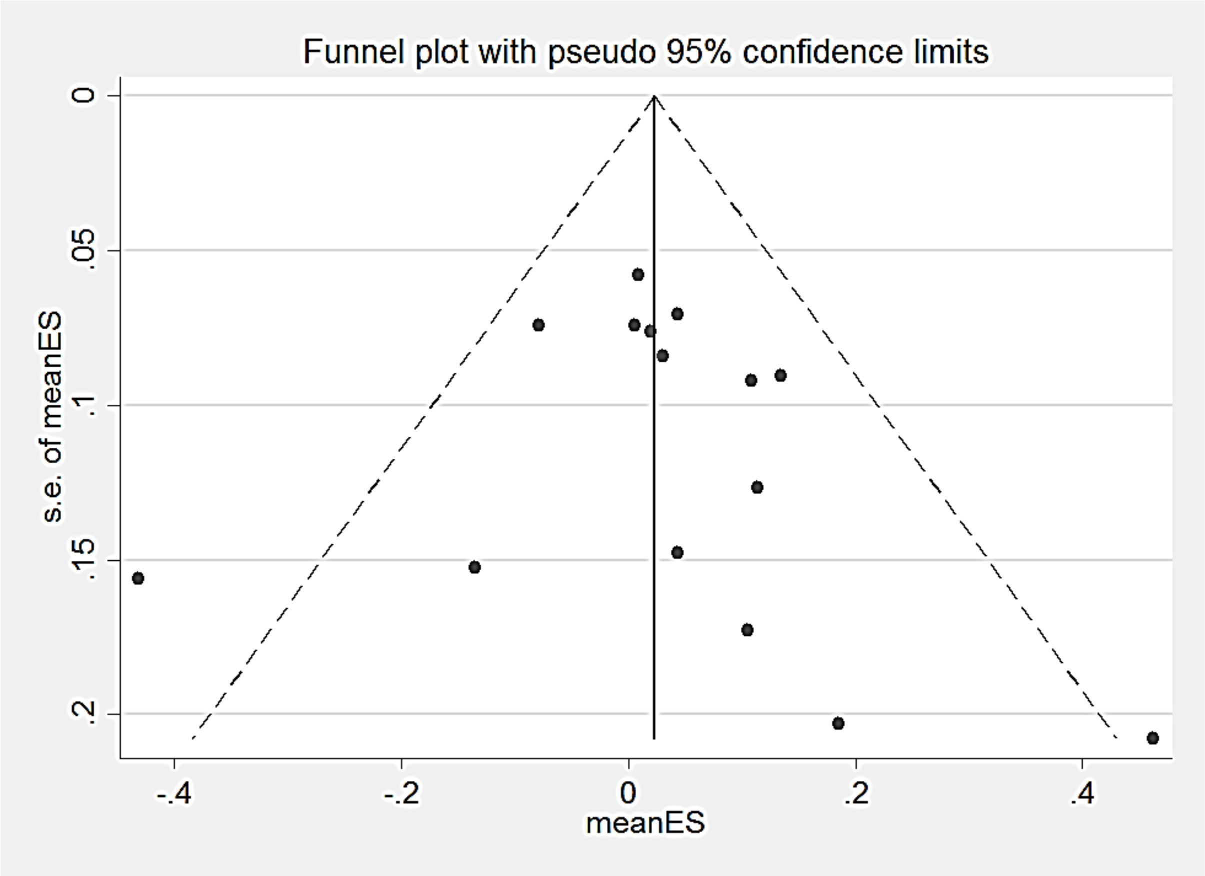

Supplementary Figure S4. Funnel plot for child motor skills outcomes with 95% confidence limits. meanSE, mean standard error.

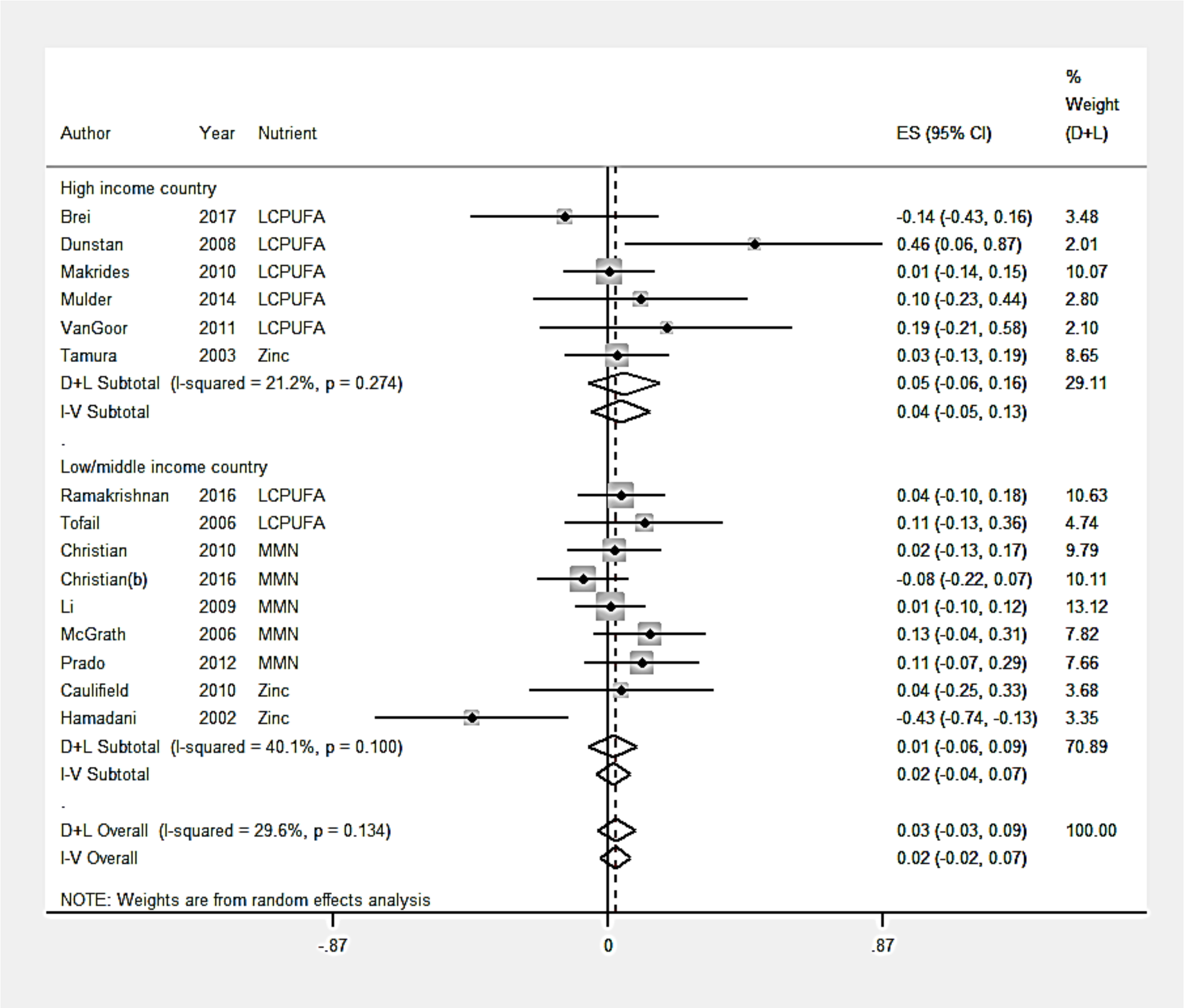

Supplementary Figure S5. Sensitivity analysis forest plot for child motor skills outcomes by country income. The overall effect size was estimated by standardised mean difference (SMD). Significance tests, high-income countries (P=0.38), low-middle income countries (P=0.70) and overall (P=0.41).

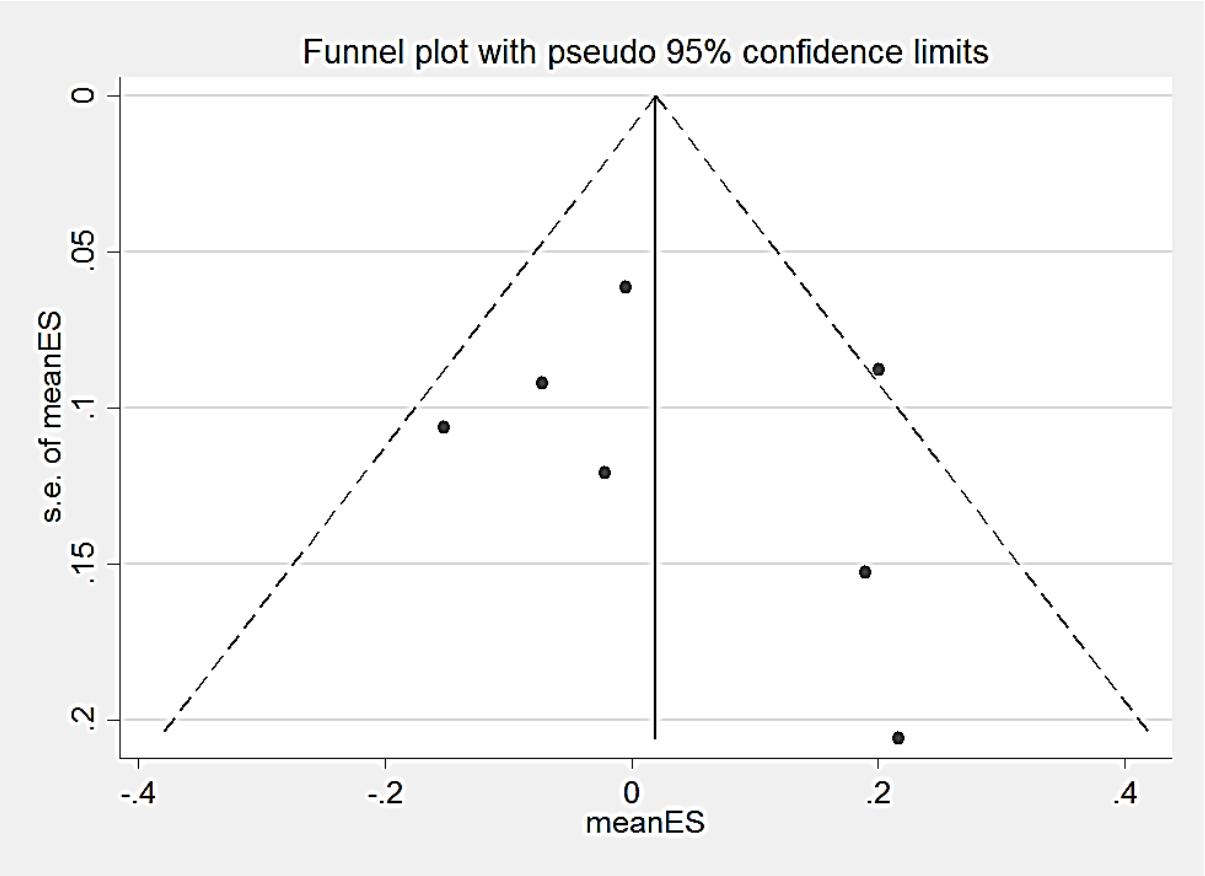

Supplementary Figure S6. Funnel plot for child fluid intelligence with 95% confidence limits. meanSE, mean standard error.

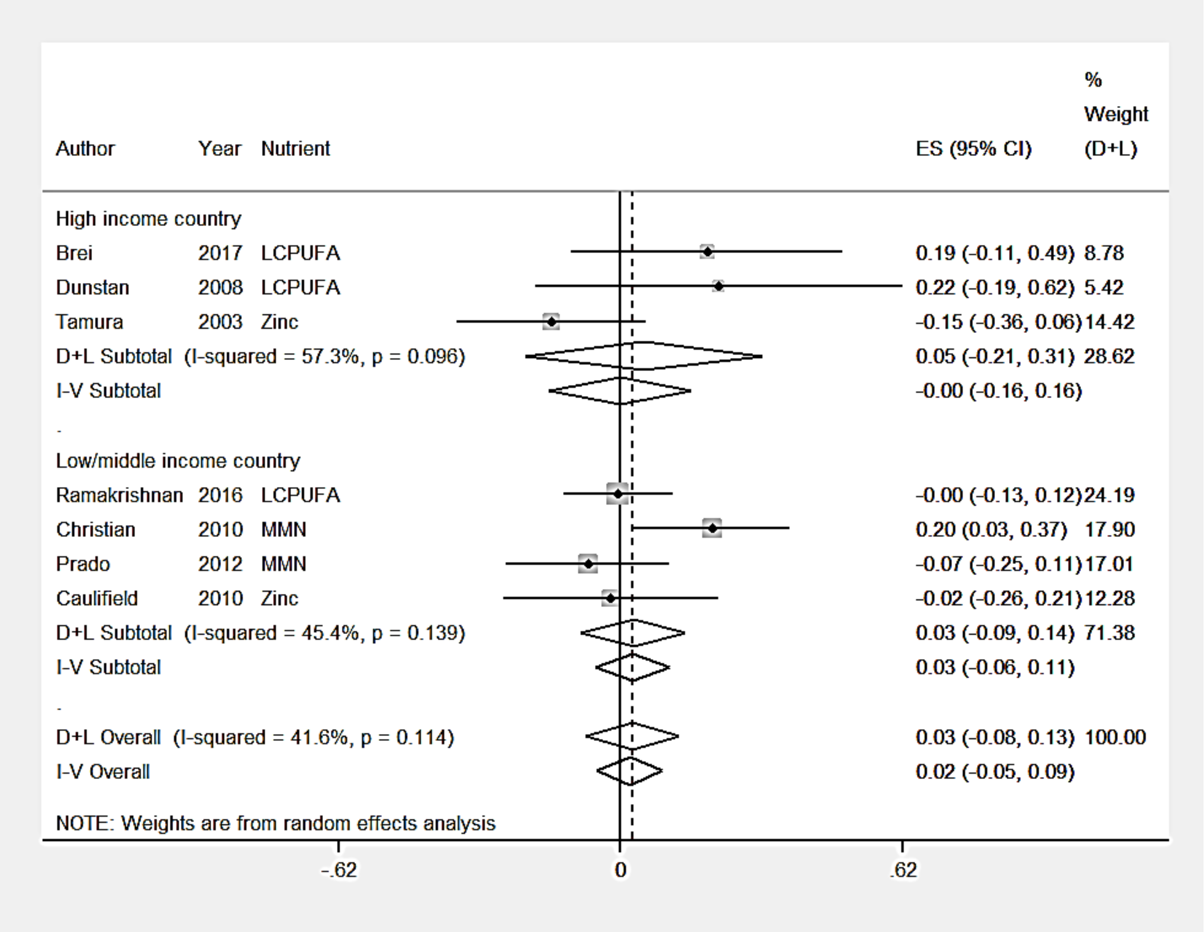

Supplementary Figure S7. Sensitivity analysis forest plot for child fluid intelligence outcomes by country income. The overall effect size was estimated by standardised mean difference (SMD). Significance tests, high-income countries (P=0.70), low-middle income countries (P=0.64) and overall (P=0.61).

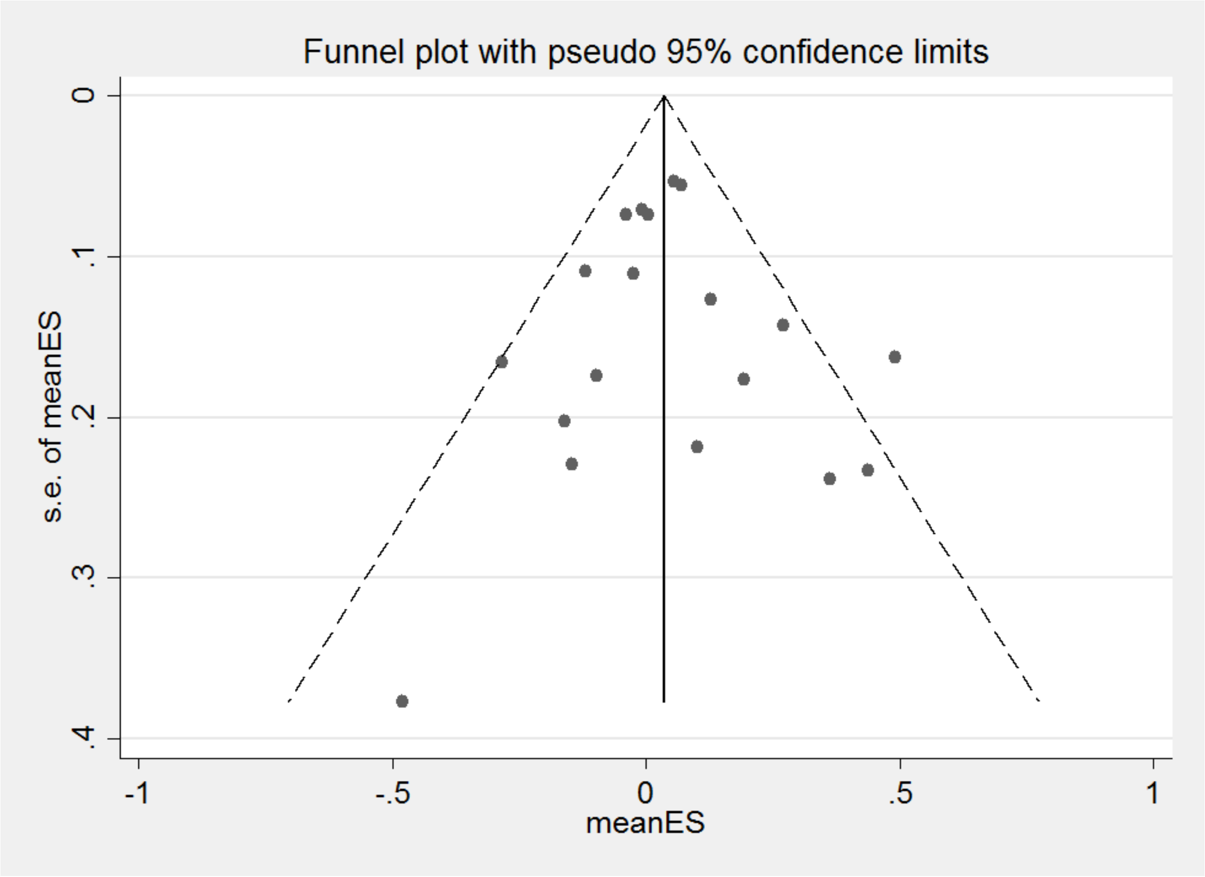

Supplementary Figure S8. Funnel plot for child global cognition outcomes with 95% confidence limits. meanSE, mean standard error.

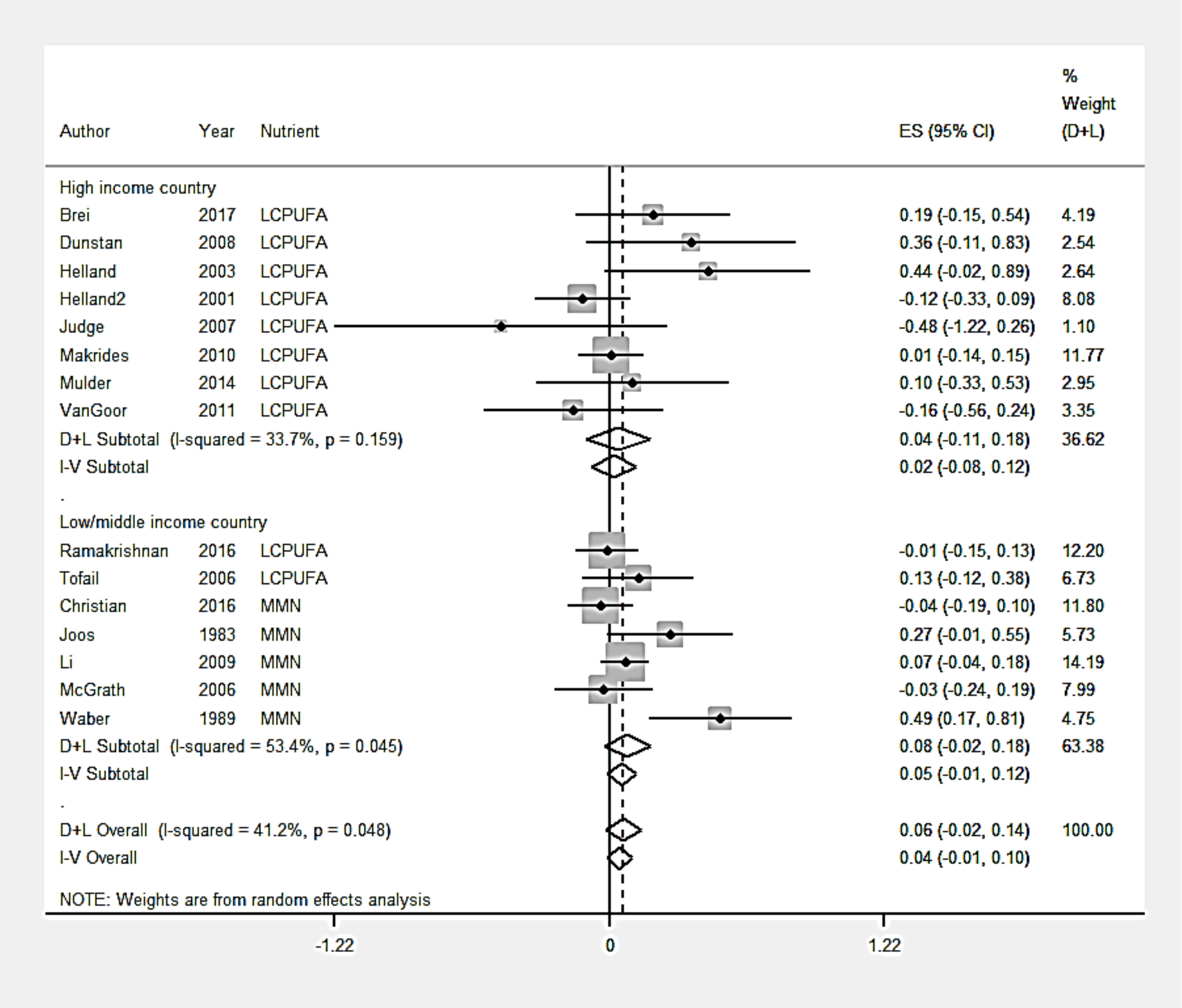

Supplementary Figure S9. Sensitivity analysis forest plot for child global cognition outcomes by country income. The overall effect size was estimated by standardised mean difference (SMD). Significance tests, high-income countries (P=0.60), low-middle income countries (P=0.13) and overall (P=0.13).

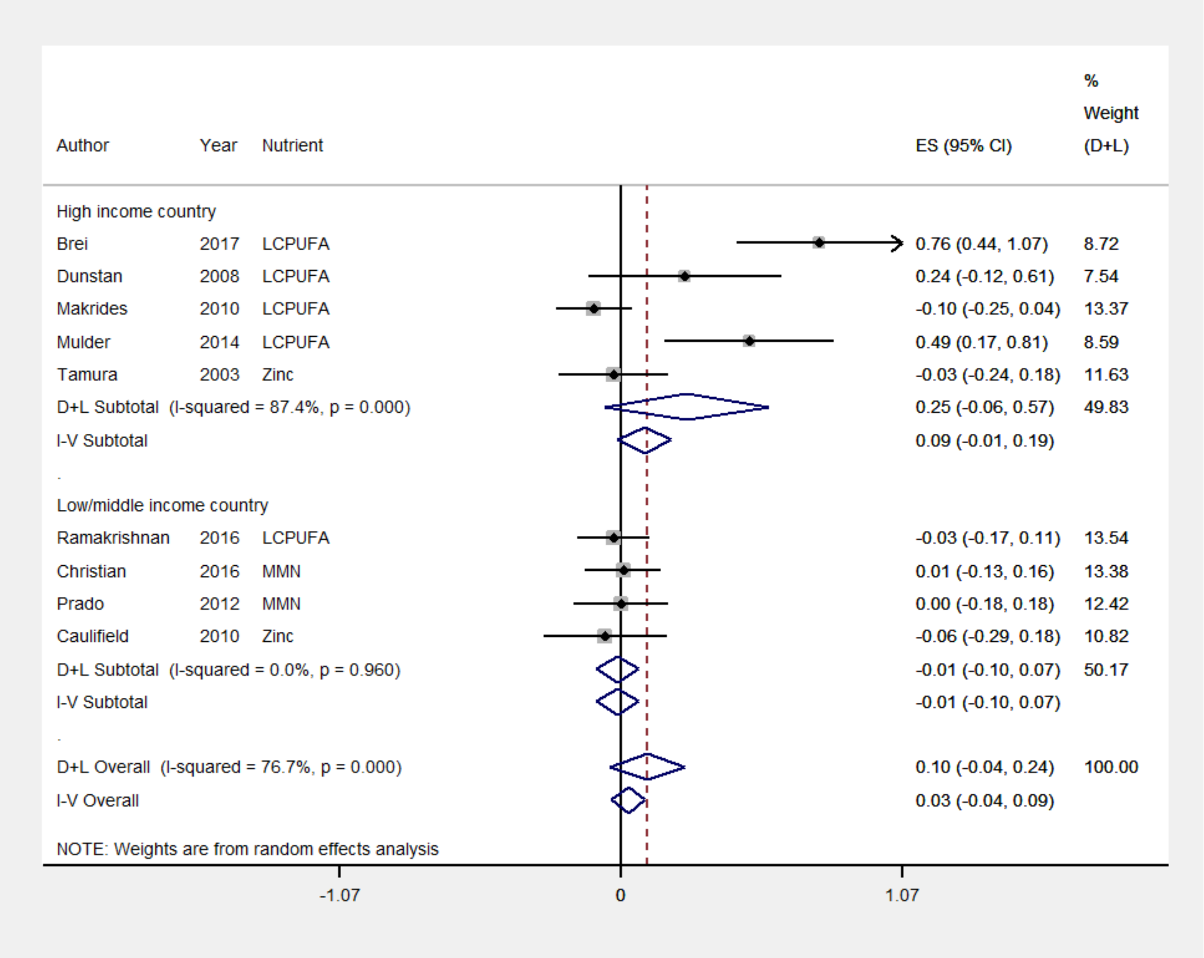

Supplementary Figure S10. Sensitivity analysis forest plot for child crystallised intelligence outcomes by country income. The overall effect size was estimated by standardised mean difference (SMD). Significance tests, high-income countries (P=0.11), low-middle income countries (P=0.76) and overall (P=0.15).
